# Engineering Liver-Specific Promoters: A Comprehensive Review of Design, Mechanisms, and Clinical Applications in Gene Therapy

**DOI:** 10.3390/cells15010014

**Published:** 2025-12-22

**Authors:** Valentin Artemyev, Anastasiia Iu. Paremskaia, Amina A. Dzhioeva, Daria Mishina, Viktor Bogdanov, Julia Krupinova, Ali Mazloum, Sofya G. Feoktistova, Olga N. Mityaeva, Pavel Yu. Volchkov

**Affiliations:** 1 Moscow Center for Advanced Studies, Kulakova Str. 20, Moscow 123592, Russia; dzhioeva.aa@genlab.llc; 2 Federal Research Center for Innovator and Emerging Biomedical and Pharmaceutical Technologies, Moscow 125315, Russia; ne.w.ay1357@gmail.com (A.I.P.); mishina.dm@genlab.llc (D.M.); bogdanov@genlab.llc (V.B.); krupinova_ua@academpharm.ru (J.K.); mazlum.a@genlab.llc (A.M.); feoktistova_sg@academpharm.ru (S.G.F.); mityaeva_on@academpharm.ru (O.N.M.); volchkov_py@academpharm.ru (P.Y.V.); 3 Moscow Clinical Scientific Center N.A. A.S. Loginov, Moscow 111123, Russia

**Keywords:** liver-specific promoters, synthetic promoters, promoters design, gene regulation, gene therapy, viral vectors, immune privilege

## Abstract

The liver is a primary metabolic hub and a pivotal target for gene therapy, owing to its capacity for protein secretion, role in metabolic homeostasis and immune tolerance. Liver-directed gene therapies are used to treat numerous inherited metabolic disorders and coagulation factor deficiencies including hemophilia (A and B), Crigler–Najjar syndrome, mucopolysaccharidoses, phenylketonuria, Fabry, Gaucher, Wilson and Pompe diseases. The efficacy and safety of liver-directed gene therapy rely on the use of strong tissue-specific promoters. To date, there are many different liver-specific promoters used in preclinical and clinical studies, including novel completely synthetic promoters. This review provides a comprehensive analysis of the design, engineering and application of liver-specific promoters. Furthermore, we discuss fundamental principles of gene expression regulation in the liver and the physiological and immunological characteristics that make it a suitable target organ for gene therapy delivery.

## 1. Introduction

The liver is a primary metabolic center in the body, regulating vital processes including metabolism (amino acids, carbohydrates, lipids, hormones, vitamins, etc.), detoxification (including xenobiotics) and the synthesis of bile, and other vital compounds. It is also responsible for the synthesis of a large number of proteins, many of which are secreted into the blood, where they mediate processes such as blood coagulation, lipid transport, and others. Thus, proteins synthesized in the liver can serve either purely intrahepatic functions (e.g., alpha1-antitrypsin, fumaroylacetoacetate hydrolase) or extrahepatic functions (e.g., factors VIII and IX) [1].

Hereditary metabolic disorders constitute a heterogeneous group of genetic diseases that primarily manifest in childhood, with a prevalence of approximately 1 in 784 newborns [2]. Many hereditary diseases, including hemophilia, hereditary angioedema, and alpha-1 antitrypsin deficiency (AATD), are associated with the secretion of defective proteins in hepatocytes, making the liver a pivotal target for treatment [3]. Metabolic diseases that require liver-targeted therapy are usually split into two main groups. The first group includes disorders associated with protein secretion in hepatocytes that indirectly affect the whole body, such as glycogen storage disease Ia, acute intermittent porphyria, Crigler–Najjar syndrome, ornithine transcarbamylase deficiency, Wilson’s disease, and others. The second group includes diseases with dysfunction of protein synthesis affected by multiple organs at once. Restoring expression of these proteins in the liver is expected to significantly attenuate disease severity. Such diseases include Fabry disease, type II glycogen storage disease, mucopolysaccharidosis types I, II, IIIA and VI [3,4].

Numerous treatment approaches have been developed for liver diseases, including cell-based, pharmaceutical, and gene therapies [3,5,6]. This review discusses in detail the design and engineering of liver-specific promoters for the development of liver-directed gene therapies for hereditary diseases, emphasizing the liver’s characteristics as a target organ, the mechanisms of gene expression regulation, and the origin and application of the main promoter systems used in liver-directed gene therapy.

## 2. Liver as a Target for Gene Therapy

Hepatocytes are the parenchymal cells of the liver representing over 80% of the liver’s volume [7]. Non-parenchymal cells represent approximately 6% of the liver’s volume and comprise hepatic macrophages (2.1%, Kupffer cells), sinusoidal endothelial cells (2.8%), hepatic stellate cells (1.5%, Ito cells), natural killers (NK) cells, cells residing in the Disse space and others [8]. Most gene therapies targeting the liver are specifically directed at hepatocytes, as they are responsible for the synthesis of the majority of proteins secreted by the liver. Even when a protein is expressed by other cell types, targeting hepatocytes is preferable due to their quantitative advantage. For example, coagulation factor VIII (FVIII) is normally expressed by liver sinusoidal endothelial cells, which are less abundant and challenging to target with vectors; therefore, gene therapy for hemophilia A primarily targets hepatocytes [9]. Thus, we refer to targeting hepatocytes in the further discussion of liver-directed gene therapies. In the following sections, we will examine the characteristics of the liver and hepatocytes as targets for gene therapies.

### 2.1. Vector Size Limitations

As a highly vascularized organ, the liver receives a large volume of blood from throughout the body, enabling viral particles introduced into the bloodstream to rapidly reach hepatic cells [10]. This fact allows the use of various vector administration methods including systemic delivery (via the tail vein), portal vein injection, and direct intraparenchymal administration, etc. [11,12,13]. However, the liver’s capillaries are lined with sinusoidal endothelial cells that contain small pores called fenestrae, which act as a filter permitting only particles smaller than the fenestrae to access parenchymal cells [14]. Consequently, there are limitations on the size of delivery vectors, especially large ones such as adenoviruses (approximately 93 nm with 30 nm fibers), when delivered to the liver via the bloodstream, depending on the model organism and liver region. The fenestrae size of human and mouse sinusoidal cells is about 100 nm (50–300 nm), and that of rabbits is 60 nm [15,16].

### 2.2. Low Hepatocyte Proliferation Rate—An Advantage and an Obstacle for Vectors

Early studies established that resection of two-thirds of hepatocytes leads to a maximal regeneration rate, which then gradually declines as regeneration proceeds [17]. Despite the well-known regenerative capacity of the liver, differentiated hepatocytes in a healthy liver are long-lived cells with low basal DNA synthesis, where approximately only 1 in 20,000 cells are undergoing mitosis at any given time [18]. Consequently, liver cells are typically in a quiescent state in vivo, which hinders nuclear entry and integration of certain retroviruses, such as Moloney’s murine leukemia virus (MoMuLV), that cannot pass through nuclear pores [19]. In some cases, liver regeneration can be induced by partial hepatectomy, but this is rarely used in gene therapy. In contrast, adeno-associated viral (AAV) vectors efficiently transduce both dividing and non-dividing cells. Therefore, the low rate of hepatocyte proliferation makes the liver a suitable target for long-term AAV-mediated gene therapy, supporting persistent and stable transgene expression [4]. An exception is found in rapidly proliferating hepatocytes during liver development. In the developing mouse liver, transgene expression declines rapidly, with stable residual expression after 2 weeks in only 4–8% of hepatocytes [20]. Thus, the low proliferation rate of hepatocytes simultaneously poses a barrier for viral vectors unable to integrate into the nucleus of non-dividing cells, yet this represents a key advantage for vectors capable of efficiently transducing quiescent cells.

### 2.3. Immune System Barriers to Liver-Directed Gene Therapy

As previously mentioned, the liver contains a large number of resident immune cells, and 90% of all resident macrophages in the body are widely represented in the liver Kupffer cells [21]. The presence of Kupffer cells in the liver poses an additional obstacle to gene therapy due to the substantial uptake of delivery vectors by these macrophages. Several studies report methods to overcome this barrier by the use of liposomes to deplete Kupffer cells [22]. Another significant problem with liver-targeted gene therapy, as with other organs, is the development of an immune response to both the expressed transgene and the delivery vector. Pre-existing immunity to delivery vectors, particularly to viral capsids, is frequently encountered Transgene expression can be significantly reduced as a result of capsid neutralization by antibodies and activation of CD8+ cytotoxic T-cells that attack transduced cells [23,24]. To overcome the neutralization of viral vectors, modifications of the capsid protein amino acid sequence and chemical modification are currently being developed [25,26]. Some recent studies have employed a novel approach to reduce the neutralization of viral particles by antibodies, which involves encapsulating AAV in a lipid nanoparticle that imitates natural enveloped viruses, helping to evade the immune response [27,28]. To mitigate the immune response mediated by activation of CD8+ cytotoxic T cells against viral capsids, it is essential to maximize transgene expression while minimizing the vector dose. Various approaches are currently used to improve the efficiency of vector-mediated transgene expression, including codon optimization of the transgene, creation of strong promoters, use of self-complementary adeno-associated viral vectors (scAAV), and transgene variants with greater activity [29,30,31]. As a general principle, the immune response to transgenes occurs more frequently in genotypes with protein reading frame alteration due to deletions, nonsense mutations, gene inversions, etc., whereas genotypes with missense mutations usually do not trigger an immune response and are characterized by more stable expression [32,33]. The use of a more efficient promoter leads to increased expression of the functional product, allowing for a reduction in the vector dose. This strategy lowers the viral capsid load delivered to hepatocytes while maintaining high transgene expression levels [34]. The balance between CD8+ T cell-mediated clearance of AAV-transduced hepatocytes and immune tolerance is dose-dependent. Specifically, a higher viral antigen load can lead to more significant immune clearance. This is associated with a downregulation of T-cell negative checkpoint markers, e.g., the programmed death 1 receptor, and upregulated expression of relevant cytokines [35]. More detailed mechanisms of transgene expression suppression related to the immune response against the viral capsid are discussed in a dedicated review [36].

### 2.4. Liver Immune Privilege in Gene Therapy

Immune privilege refers to the status of an organ in which the presence of antigens does not trigger an inflammatory immune response. Typically, immune-privileged organs include the brain and central nervous system, eyes, and the pregnant uterus. In addition to limited regenerative capacity, features of immune privilege include restricted antigen drainage to lymph nodes, low expression of MHC I, the elimination of inflammatory cells that enter the organ (e.g., through the FasL pathway), and immune deviation (such as ACAID and BRAID) [37]. Recently, other organs and tissues, including the testes, liver, hair follicles, and even the intestinal mucosa, as well as tumors, are also considered immune-privileged [38]. The liver has a special form of immune privilege called hepatic tolerance, where liver transplantation leads to donor-specific T-cell tolerance [39]. This phenomenon was demonstrated in an early study on allogeneic liver transplantation in pigs without prior immunosuppression [40].

The liver serves a barrier function and produces numerous neoantigens; therefore, the risk of immune response activation in the liver is very high. To moderate this, the liver employs several key mechanisms of immune tolerance:

1. Non-parenchymal liver cells (including stellate cells and plasmacytoid dendritic cells) produce a large number of immunosuppressive anti-inflammatory cytokines, such as IL-10, TGF-beta [41];
2. Liver natural killer cells express a negative T-lymphocyte costimulatory–the programmed cell death ligand (PD-L1) [42];
3. Hepatocytes themselves also contribute to immune tolerance by producing PD-L1 [43].

Another aspect of the special liver immune privilege is induced immune tolerance to antigens introduced either directly into hepatocytes or systemically through the portal vein [39]. It is suggested that regulatory CD4+CD25+ Tregs and the tolerogenic properties of the liver, including the extensive expression of anti-inflammatory cytokines such as IL-10 and TGF-beta, play a central role in this mechanism. These cytokines suppress both antibody and T-cell immune responses to the endogenously expressed transgene [44,45]. Vector delivery to the liver results in lower titers of neutralizing antibodies compared to the previously common muscle-directed delivery. This is supported by studies in animal models of hemophilia. Blood coagulation factor IX (FIX) gene transfer to mice and dogs via intramuscular injection of AAV vectors led to rapid inhibitor formation and was effective only in combination with immunosuppression [46]. In another study, AAV-FIX delivery via the mesenteric and portal veins provided a lower level of neutralizing IgG production or its absence in some cases [47].

It was also demonstrated that AAV-mediated liver-directed gene therapy was successfully delivered to mice pre-immunized with FIX protein and thus possessing antibodies against it. This approach resulted in long-term disease correction and a dramatic reduction in antibody titers, even with the repeated protein presentation [9,48]. This strategy was also applied to other metabolic disorders and lysosomal storage diseases [49,50]. Currently, the immune tolerance induction to a transgene delivered to the liver is just beginning to be studied in clinical trials.

Successful induction of immune tolerance in animal models has enabled the first human trials to be conducted [48,51]. BMN-270 Phase I/II clinical trial by BioMarin Pharmaceutical was started in 2020 and subsequently showed promising preliminary results in the treatment hemophilia A patients with pre-existing immunity to coagulation factor VIII (FVIII) [52]. Two patients with a history of FVIII inhibitors and two patients with active inhibitors received AAV-mediated liver-directed gene therapy for endogenous FVIII expression. In patients with a history of FVIII inhibitors, no antibody reappearance occurred, and the treatment proved effective. In one patient with active FVIII inhibitors a decrease in inhibitor titers was observed, along with an increase in FVIII concentration and activity in the blood by week 28 [53]. The therapy is currently known as Valoctocogene roxaparvovec (Roctavian). Thus, targeting transgene expression to the liver not only provides therapy but also induces specific immune tolerance to the transgene, which is particularly important for patients with pre-existing immunity. To sum up, liver-directed delivery is a promising approach to reduce the immune response to the transgene due to the special immune privilege of the liver as an organ.

## 3. Liver Gene Expression Regulation

### 3.1. Genomic Regulatory Elements

Gene expression is controlled by regulatory elements, including promoters, enhancers, insulators, and silencers, which can interact with transcription factors (TFs) and co-regulators [54,55]. In gene therapy, a promoter usually refers to a promoter construct–a combination of regulatory elements sufficient to drive transgene expression. Promoter activity is typically quantified as the level of gene expression in terms of mRNA and protein, with the assumption that higher mRNA/protein levels correspond to greater promoter activity. Increasing promoter activity is key to the effectiveness of liver gene therapies, so it is important to consider the mechanisms that regulate them. Increased transcription levels can be achieved by targeting regulatory elements that recruit transcription factors or cofactors and promote pre-initiator complex stabilization, accelerating transcription initiation [56].

**Figure 1:**
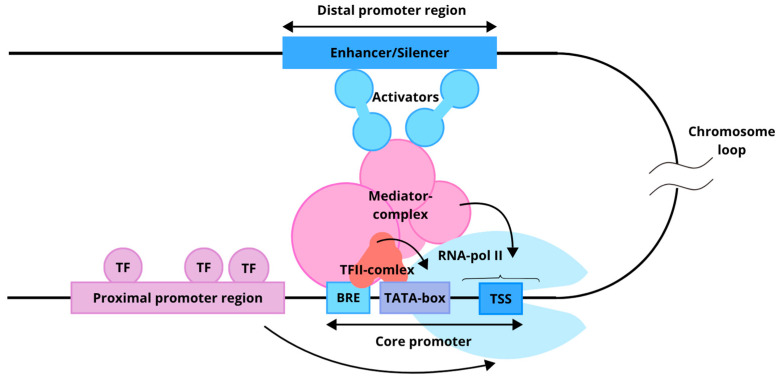
Functional model of a eukaryotic promoter. The core promoter recruits a set of general transcription factors (TFII complex) and RNA polymerase II. The proximal promoter region, composed of distinct regulatory elements, binds additional transcription factors that either enhance or suppress transcription. The distal region, represented by an enhancer or silencer, can be located several thousand nucleotides away from the core promoter. Arrows indicate the influence of the proximal and distal regions on the core promoter.

In molecular biology, promoters are DNA regions located near transcription start sites (TSS), where transcription initiation occurs (Figure 1). In a natural eukaryotic promoter, two main regions are distinguished: the core promoter (minimal promoter) and the proximal promoter [54,57].

The core promoter is a set of sequences sufficient for assembling the pre-initiation complex, which includes RNA polymerase II and associated general transcription factors (GTFs) [57]. In vitro localization of the core promoter is sufficient to determine the TSS. The structure of a minimal promoter and the set of sequences it contains are diverse. Common and well-known regulatory sequences present in a core promoter include the Inr (initiator sequence), TATA box, BRE (TFIIB recognition element), DPE, and others [58]. The binding of GTFs (TFIIA, TFIIB, TFIID, TFIIE, TFIIF, and TFIIH) enables the core promoter to initiate transcription, although it typically exhibits low basal activity [59]. Located upstream of the core promoter, the proximal promoter spans several hundred nucleotides and contains regulatory elements that bind transcription factors (TFs). These TFs can either repress or enhance transcription, thereby modulating core promoter activity [55].

The combination of regulatory elements and the availability of cell-type-specific TFs determine promoter specificity. Promoters are thus broadly categorized as tissue-specific or ubiquitous. Tissue-specific promoters, such as hAAT (human alpha-1 antitrypsin) (liver) or MCK (muscle), facilitate targeted expression in specific tissues or cells, and their function directly depends on the presence of TFs in the respective tissue. Ubiquitous promoters, such as cytomegalovirus (CMV) immediate early promoter, chicken β actin promoter, and promoter of ubiquitin C, drive widespread expression across all tissue [60]. Additionally, promoters are traditionally classified as either constitutive or inducible. Constitutive promoters maintain a constant level of gene transcription, independent of specific external signals. This type of regulation is typical for genes encoding proteins involved in fundamental cellular processes. The expression of genes under an inducible promoter depends on some external chemical or physical stimuli [61].

An enhancer is a distal regulatory region that modulates transcription levels from a promoter, irrespective of distance relative orientation, as their interaction is facilitated by the three-dimensional architecture of chromatin [62,63]. Enhancer sequences can be identified by chromatin modifications, including histone modifications [64] as well as by bidirectional transcription of enhancer RNA from these regions [54,65,66]. Enhancers increase gene expression through two complementary mechanisms involving chromatin architecture and specific motif organization: At the chromatin level, enhancers overcome the energetic barrier of nucleosomes through cooperative binding of multiple transcription factors with proper spacing, orientation, and positioning (the “enhancer grammar”), which recruit coactivator complexes (p300/CBP, SAGA, Mediator); these coactivators perform acetylation and methylation of histones (H3K27ac, H3K4me1) and recruit chromatin-remodeling factors that physically displace nucleosomes, opening DNA access for RNA polymerase II. The number of transcription factor motifs positively correlates with nucleosome eviction intensity and gene expression output, following either rigid “enhanceosome” architecture (with fixed spacing and orientation) or flexible “billboard” models (with variable motif arrangement), with most enhancers occupying a spectrum between these extremes. At the three-dimensional level, enhancers establish spatial contacts (loops) with target gene promoters within topological associated domains (TADs), directing recruited coactivators and transcription factors to transcription initiation sites and enhancing RNA polymerase II recruitment and transcriptional burst frequency. Furthermore, multiple enhancer copies (“shadow enhancers”) can function additively, synergistically, or competitively, providing regulatory robustness and ensuring precise, reproducible gene expression levels across developmental and evolutionary contexts [67].

Furthermore, more than 1000 liver genes are expressed under the control of super-enhancers, including TFs (*HNF4A*, *C/EBPa*, and *HNF1B*), cytochromes (*CYP2E1* and *CYP8B1*), albumin (*ALB*), blood coagulation factors, and others [68]. A super-enhancer is a cluster of enhancers characterized by a high density of TFBS, active chromatin marks (H3K4me1, H3K27ac, and P300), and association with the transcription activators Mediator Complex Subunit 1 (MED1) and Bromodomain-containing Protein 4 (BRD4). These elements are located at a considerable distance from each other but within a range not exceeding 12.5 kb [69,70]. The Mediator complex performs multiple functions during transcription, with its primary role being the transmission of regulatory signals from enhancer regions to the transcriptional machinery through activators [71]. In addition, the complex regulates chromosomal spatial architecture by supporting three-dimensional contacts between the core promoter and distant regulatory regions [72]. MED1 and BRD4 participate in the formation of biomolecular condensates and promote the assembly of dynamic transcriptional complexes through liquid–liquid phase separation (LLPS) [73]. LLPS is a physicochemical process in which molecules organize into dense and dilute phases. The phase transition occurs abruptly, providing a key functional property of super-enhancers—the ability to rapidly and stably activate gene expression in response to minor fluctuations in transcription factor concentration [70,74]. It should be noted that groups of enhancers regulating the expression of the same gene are characterized by functional redundancy [75,76], meaning they perform similar or overlapping functions. This mechanism ensures the robustness of transcriptional regulation, preventing major changes in expression upon the loss or disruption of activity of one of the elements. Moreover, super-enhancers have been shown to play an important role in the regulation of microRNA transcription as well as in their maturation, including the processing of primary microRNAs [77].

Transcription factors play a key role in the development and maintenance of cellular identity, normal cell function, under stress conditions, and during the development of pathological processes. A group of pioneer TFs is distinguished by their ability to bind to compacted (“closed”) chromatin, promote the remodeling of adjacent regions, and increase their accessibility to other TFs. The structure of TFs varies, but in most cases, they include several functional domains. Among them are a domain responsible for the specific recognition of DNA sequences and an effector domain that modulates the expression of the target gene. Typically, transcription factor-binding sites (TFBS) are enriched in promoter and enhancer regions [78]. The direction of gene expression regulation (activation or repression) is determined by the availability of co-activators or co-repressors with which a TF can interact. For example, the interaction between SIRT6 and FOXA2 inhibits the activity of the Zeb2 promoter in hepatocellular carcinoma cells [79]. In contrast, a study using a mouse model showed that the PGC-1β–FOXA2 complex enhances the transcription of genes encoding mitochondrial β-oxidation enzymes (CptI, Mcad, and Vlcad) [80]. Pioneer TFs are often less selective and can form paired interactions with a large number of TFs available in the cell.

Some TF pairs do not interact directly, but are capable of cooperatively binding to neighboring DNA sites and ensuring joint gene regulation. In most cases, such interaction is typical for pioneer TFs. For example, analysis of the ALB enhancer has shown that the cooperative binding of FOXA1 and GATA4 increases chromatin accessibility by repositioning nucleosome N1 at the NS-A1 and eF sites [81]. According to the affinity theory proposed by Zhao et al., TFs exhibit varying degrees of pioneer activity, determined by their ability to bind DNA [82]. In their study, the authors demonstrated that FOXA1 and HNF4A can regulate the expression of liver-specific genes both independently and cooperatively. Notably, the density of TFBS is higher in closed chromatin regions, which likely reflects the need to overcome greater energetic barriers to initiate transcription.

The spatial organization of regulatory elements and their accessibility for binding TFs play a critical role in the regulation of gene expression. In this regard, the identification of natural regulatory sequences and the design of synthetic ones represent important areas of research in molecular and synthetic biology. To develop gene therapies targeting the liver, key components of the liver’s transcriptional regulatory network are being studied. The main transcription factors regulating gene expression in the liver, as well as their molecular interactions, will be discussed below.

### 3.2. Liver-Enriched Transcription Factors

Hepatocyte nuclear factor 4 alpha (HNF4A) is a member of the nuclear receptor superfamily, covering cis-regulatory regions of at least 42% of actively transcribed genes in hepatocytes and binding to DNA as a homodimer [83]. This TF is involved in the regulation of many liver functions, participating in the metabolism of xenobiotics, bile acid synthesis, lipid homeostasis, gluconeogenesis, cell proliferation, apoptosis, etc. HNF4A is involved in establishing and maintaining active chromatin marked by H3K4me1 and H3K4me3 signatures [84]. The *HNF4A* gene is encoded on chromosome 20 and regulated by two promoters, P1 and P2. Alternative splicing from P1 generates six isoforms (HNF4A1-6), which are characteristic of normal adult liver and kidney tissues, while six isoforms from the P2 promoter (HNF4A7-12) are expressed normally in fetal liver, pancreas, and in adult liver in response to starvation, as well as in various pathological conditions. The role of HNF4A in the cellular identity of hepatocytes is interesting: P1-HNF4A acts as a tumor suppressor, whereas increased expression of P2-HNF4A is associated with the progression of hepatocellular carcinoma [85]. Both isoform groups are also expressed in the colon epithelium and pancreatic ducts, where an increase in P2 isoforms is associated with potential cancer progression [86]. HNF4A consists of five structural domains: the N-terminal A/B domain, the C domain, which includes the functional DNA-binding domain, the D and E domains that form the ligand-binding domain, and the C-terminal F-domain. The N-terminal and C-terminal domains have a disordered structure and do not participate in DNA motif binding; however, they contribute to the differences between isoforms [87,88]. Interacting with co-regulators, HNF4A can exhibit both repressive and activation activities. For example, the interaction with PGC1α [21], SRC-1, and GRIP1 [89], as well as PPARα, significantly enhances transcription of the target gene. In contrast, IRF2BP2 [90] and DAX-1 [91] act as co-repressors. HNF4A predominantly exerts its activity during the night, directly regulating the circadian expression of downstream genes by acting as a repressor of the CLOCK:BMAL1 heterodimer [92,93].

Hepatocyte nuclear factor 1 alpha (HNF1A) is a transcription factor from the Pit–Oct–Unc (POU) family, encoded on chromosome 12. The protein consists of several domains: a N-terminal dimerization domain, an acidic amino acid region, a POU homeodomain that includes two subdomains, POUs and POUh, and a C-terminal transactivation domain [94]. HNF1A regulates the expression of *HNF4A* through an auto-regulatory positive feedback loop [95]. Somatic mutations in the POU domain lead to reduced transcriptional activity of the P1-HNF4A and P2-HNF4A isoforms, which is associated with decreased promoter affinity [96]. HNF1A is involved in lipid metabolism, maintaining cholesterol homeostasis through the regulation of PCSK9 and miR-122-dependent activation of SREBP-2 [97]. It inhibits the expression of SREBP-1c and the activity of the STAT3 signaling pathway [98], thereby preventing lipid accumulation in the liver. Moreover, HNF1A is associated with MODY3, as it normally enhances IRS-1 and AKT phosphorylation and activates the insulin signaling pathway [96]. HNF1A regulates the expression of complement pathway components such as C5, C8A, and factor D [99,100]. A decrease in hepatic HNF1A expression during fibrosis leads to the activation of inflammatory signaling pathways, including NF-κB and JAK/STAT, which in turn form a positive feedback loop that further suppresses HNF1A [101,102]. Thus, HNF1A represents one of the key factors involved in lipid and carbohydrate metabolism as well as in modulating the hepatic immune response.

Prospero-related homeobox 1 (Prox1) is a transcription factor that plays a critical role in liver development and regeneration, promoting the recovery of the hepatocyte population [103,104,105]. It is required for the migration and differentiation of hepatocytes and cholangiocytes during organogenesis. This factor can directly repress the expression of genes unrelated to liver differentiation, thereby helping to maintain the hepatocyte phenotype [106]. The regulatory role of Prox1 as a tumor suppressor has been discussed in detail in a recent review by Lee and Ma [107]. Genes under its regulatory influence also include other hepatic transcription factors, as illustrated by HNF4A [108]. In addition to direct Prox1 binding, an interaction has been demonstrated between the N-terminal LXXLL motif of Prox1 and the activation function 2 domain of HNF4A [109].

Members of the Forkhead box (FOX) transcription factor subfamily, FOXA, also known as HNF3, constitute a group of pioneer transcription factors and include three main proteins: FOXA1, FOXA2, and FOXA3. FOXA is expressed in various tissues, including the liver, pancreas, intestine, lungs, and prostate. FOXA3 regulates bile acid transport by inhibiting the NTCP and OATP1 transporters, preventing excessive lipid accumulation in the liver, and suppressing inflammatory responses through inhibition of TNF-α, interleukin-1β, Icam1, Nfκb1, Cd68, and Jnk1. FOXA3 and HNF4A co-regulate the cell cycle via activation of the TP53 gene promoter [110,111,112], accompanied by increased histone H3K9 acetylation at the promoter region [110]. FOXA1 and FOXA2 have partially overlapping functions and play key roles in the development and maintenance of hepatocyte identity [113,114,115]. Literature data indicate that their knockdown reduces the expression of major hepatic transcription factors and redirects the cellular phenotype toward alternative differentiation trajectories, showing increased neuroectodermal and pluripotency markers [116]. 

The ONECUT (OC) transcription factor family, also known as HNF6, exists in three isoforms: OC-1 (HNF6A) and OC-2 (HNF6B), which are predominantly expressed in the liver, and OC-3, which is less studied but primarily expressed in neural tissue. HNF6 has been extensively reviewed in a recent publication [117]. In this work, we focus only on the main aspects of its regulatory activity. HNF6 is known to interact differently with the promoters of *TTR* and *FOXA2*. In the *FOXA2* promoter, only the cut-domain binds DNA, while its LSDLL motif and F48M50 dyad of the homeodomain are available to interact with the co-activator CREB-binding protein (CBP). In contrast, in the *TTR* promoter, binding involves both domains of HNF6A: the cut domain and the homeodomain, recruiting the co-activator P300/CBP-associated factor. Notably, changing only two nucleotides in the DNA motif leads to such a substantial alteration in the activation mechanism [118].

The transcription factor Activator Protein-1 (AP-1) binds DNA as a dimer. The components of this dimer can include proteins from various families, such as JUN (c-Jun, JunB, and JunD), FOS (c-Fos, FosB, Fra-1, and Fra-2), MAF (musculoaponeurotic fibrosarcoma) protein family, and activating transcription factor (ATF) [119]. The composition and functional outcome of AP-1 TFs depend on upstream signaling pathway activity (e.g., MAPK and JNK) and cytokine availability. For example, interleukin-6 (IL-6) induction enhances the transcription of insulin-like growth factor-binding protein 1 through the HNF-1 site via AP-1 (c-Fos/c-Jun) and STAT3 [120]. A similar mechanism, AP-1/HNF-1, increases the expression of C-reactive protein [121]. Thus, AP-1 TFs are involved in cellular responses to oxidative stress [122], inflammation [123], and high-fat intake [124]; AP-1 TFs can also induce apoptosis [125] and regulate proliferation [126].

The C/EBP family of transcription factors can form homo- or heterodimers and bind DNA via a leucine zipper domain. Members of the C/EBP family are expressed in many tissues; however, in the liver, the primary regulatory roles are carried out by two transcription factors: C/EBPa and C/EBPb. C/EBPa exists in several isoforms: the full-length p42, the truncated p30, and an extended isoform, expressed at lower levels, which initiates translation from an alternative start codon (CUG in vertebrates, GUG in humans) [127].

C/EBPb, also known as interleukin-6-dependent DNA-binding protein or nuclear factor interleukin-6 (NF-IL6) [128], is also represented by three isoforms: LAP1 and LAP2, which generally act as transcriptional activators, and the shorter LIP isoform, which more often functions as a transcriptional inhibitor. C/EBPa exhibits anti-proliferative effects [129] and is involved in the regulation of lipogenesis [130] and gluconeogenesis through the co-activator CBP [131]. Overexpression of C/EBPa in hepatic stellate cells increases the expression of ATG5 and Beclin1, thereby promoting autophagy [132].

C/EBPb is induced by IL-6 and plays a key role in the regulation of immune responses [133]. In addition, this transcription factor acts as an effector of endocrine stimuli, particularly thyroid-stimulating hormone (TSH). TSH increases the expression of miR-374b, which subsequently suppresses C/EBPb transcription and directly targets the 3′-untranslated region of C/EBP, creating a negative feedback loop [134].

It should be emphasized that the regulatory activity of TFs is determined by several interconnected factors. First, it is constrained by the ~150 bp span of nucleosomes and exhibits a periodicity of approximately 10.5 bp, which reflects the helical turns of DNA. TFBS affinity also strongly influences specificity: cooperative effects of low-affinity sites can promote tissue specificity, whereas high-affinity sites may reduce it. DNA-mediated cooperativity is frequently observed; in this case, adjacent sites and defined spacing between them stabilize simultaneous binding. This property is used in the design of synthetic promoters (as described in the corresponding section). The mechanisms of cooperative interactions are discussed in greater detail in the review [135].

In addition, the composition and arrangement of TFs on enhancers and promoters can be flexible, reflecting diverse mechanisms of cooperativity and enabling TFs to function across different regulatory contexts. Thus, understanding the regulatory landscape is essential for the development of applied solutions in gene therapy.

## 4. Safety Advantages of Liver-Specific over Ubiquitous Promoters

Most gene therapy studies and commercial genetic platforms utilize natural promoters within their delivery constructs. These include ubiquitous and tissue-specific eukaryotic promoters, as well as viral promoters [58]. Numerous gene therapy studies, in particular, employ viral promoters such as the early CMV promoter [136], the simian virus 40 (SV40) promoter [137], the adenoviral major late promoter (MLP) [138], and various retroviral long terminal repeat (LTR) promoters [139]. In their native context, strong viral promoters are essential for efficient viral replication and therefore often drive substantially higher transcription levels than native eukaryotic promoters. Moreover, they are generally more compact than eukaryotic counterparts, making them easier to manipulate and utilize in gene therapy vectors [29]. In addition to natural viral promoters, synthetic ubiquitous promoters such as CBA and CAG are widely used; these contain both viral and eukaryotic regulatory elements. A comprehensive review on the clinical landscape of AAV-based gene therapies reported that between 2015 and 2019, 45% of clinical trials employing constructs with disclosed promoters used CMV, CAG, or CBA promoters [140].

The primary limitation of using viral promoter use in gene therapy is their propensity to trigger immune activation, resulting in rapid decline in transgene expression. Silencing of viral promoters is partly associated with the induction of pro-inflammatory cytokines, such as TNF-α (tumor necrosis factor-alpha) and IFN-γ (interferon-gamma), during the activation of the innate immune response [29,141]. Several studies suggest that inhibition occurs at the level of transgenic mRNA, and cytokines do not cause degradation of vector DNA or suppress general cellular protein synthesis. The analysis indicates that IFN-γ and TNF-α lead to promoter-specific, post-transcriptional destruction of viral mRNA. It was demonstrated that viral promoters are more sensitive to the inhibitory action of inflammatory cytokines compared to eukaryotic promoters [142]. An early study reported that expression of factor IX under the elongation factor 1α (EF1α) promoter in mouse liver persisted for at least six months, whereas expression driven by the CMV promoter was eliminated by week 5 [143].

Another issue associated with the use of viral regulatory elements, particularly those of retroviral origin, is their targeted methylation in eukaryotic cells, which inhibits transgene expression [144]. Viral promoters (e.g., CMV) are rich in CpG dinucleotides, which makes them targets for DNA methyltransferases and leads to epigenetic silencing. In contrast, many endogenous liver-specific promoters have low CpG content or protected chromatin contexts, which provides them with resistance to hypermethylation and more stable expression [145]. This mechanism is discussed in detail in the another review [146]. It was also reported that the epigenetic status of the transduced cell with AAV vectors effects transgene expression [147].

An alternative solution was to use eukaryotic ubiquitous promoters such as EF1α and PGK. However, ubiquitous eukaryotic promoters also present significant challenges, including the difficulty in achieving physiological expression levels and the risk of ectopic expression in non-target cells and tissues. Ectopic transgene expression often leads to adverse outcomes such as inflammation and other immune responses associated with de novo expression in non-privileged tissues and antigen-presenting cells [148].

In contrast, such as ApoE/HCR1-hAAT (human alpha1-antitrypsin promoter coupled with hepatic control region 1 of apolipoprotein gene cluster), and TBG (thyroxine-binding globulin promoter), provide higher transgene expression levels compared to ubiquitous eukaryotic and viral promoters, and do not lead to the transgene-specific antibodies formation [149,150].

Thus, a crucial requirement in the development of effective and safe gene therapies is not only the level of expression but also its localization within target tissues. The selection of a suitable promoter for targeted expression becomes particularly important, and tissue-specific promoters are increasingly being used in gene therapy development, as supported by recent data [140]. Therefore, the following section will focus on the most commonly used liver-specific promoters in gene therapies and the key stages in the creation of their improved synthetic variants (Table 1, Supplementary Material S1). More information about liver-directed gene therapies are presented in Supplementary Table S1.

## 5. Promoters Based on Human *SERPINA1* Gene

Alpha 1-antitrypsin (AAT) is the main inhibitor of serine proteases, encoded by the *SERPINA1* (Serpin Family A Member 1) gene approximately 12.2 kb in length. The healthy plasma AAT level is between 0.9 and 2 g/L, but it can be increased fivefold relative to normal during an acute-phase response (APR) [233]. AAT is expressed in the various isoforms and is controlled by two promoters for different tissues. The promoter at the 5′-end of the gene with TSSs in the untranslated exon 1A drives expression in monocytes, macrophages, and lungs, while the liver-specific promoter is located closer to the coding region with a TSS mapped in the untranslated exon 1C [234,235]. Since in this paper we discuss the use of promoters for liver-specific expression, we will focus on describing the *SERPINA1* promoter with TSS in exon 1C, and will use base numbering relative to the corresponding TSS.

**Figure 2:**
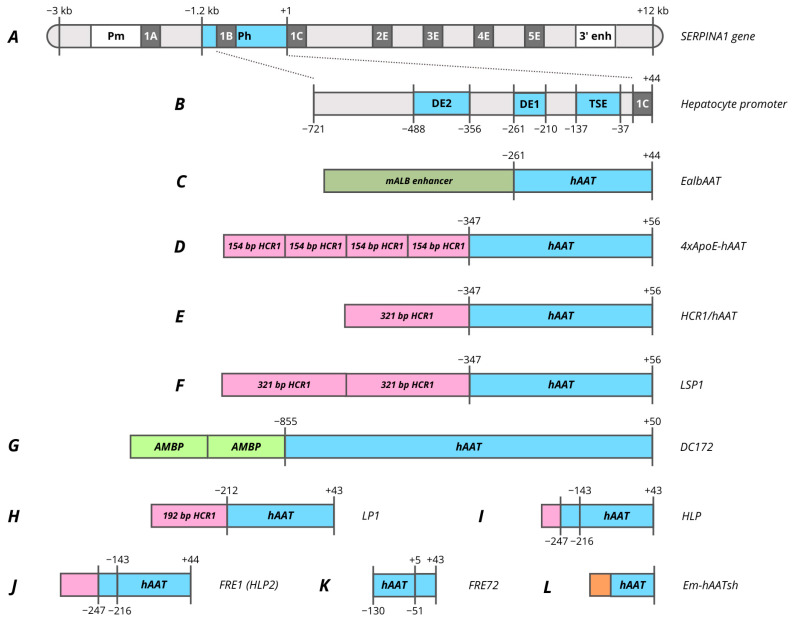
Promoters based on human SERPINA1 (AAT) gene. (**A**)—human SERPINA1 gene. (**B**)—−721/+44 hAAT promoter. (**C**)—EalbAAT promoter composed of hAAT promoter with murine Alb enhancer. (**D**)—4xApoE-hAAT promoter composed of hAAT promoter with four copies of 154 bp HCR1 enhancer. (**E**)—HCR1-hAAT promoter composed of hAAT promoter with 321 bp HCR1 enhancer. (**F**)—LSP1 promoter composed of hAAT promoter with 2 copies of 321 bp HCR1 enhancer. (**G**)—DC172 promoter composed of hAAT promoter with 2 copies of 160 bp AMBP enhancer. (**H**)—LP1 promoter composed of hAAT promoter with 192 bp HCR1 enhancer. (**I**)—HLP promoter composed of two hAAT promoter parts with 34 bp HCR1 enhancer. (**J**)—FRE1 (HLP2) promoter composed of two hAAT promoter parts with 117 bp HCR1 enhancer. (**K**)—FRE72 promoter composed of two hAAT promoter parts. (**L**)—Em-hAATsh promoter composed of four hAAT promoter parts with synthetic Em enhancer. Pm, monocytes and macrophages promoter; Ph, hepatocytes promoter; 1A, 1B and 1C, untranslated exons; 2E, 3E, 4E and 5E, translated exons; 3′ enh, enhancer located downstream from coding sequence; DE2, distal element 2; DE1, distal element 2; TSE, tissue-specific element.

In the 1980s, sequencing of the coding and non-coding regions of the *SERPINA1* gene (formerly termed *AAT*) enabled a detailed studies of its promoter regulation [236,237]. Subsequent studies identified multiple transcript variants of this gene and two principal promoters located approximately ~2 kb apart. The upstream promoter is responsible for expression in macrophages, while the downstream promoter provides expression in hepatocytes (Figure 2A) [238]. The inducibility of the promoter is determined by the action of cytokine IL-6, mediated through transcription factor NF-IL6, whose binding site is located in the 3′ enhancer. The 3′ enhancer region also contains AP1 sites for binding to Fox/Jun and an Oct-1 binding site. IL-6 induction has minor effect on the 5′ promoter separately from the 3′ enhancer, suggesting complex spatial regulation of inducible promoter activation [238,239,240].

However, the 5′ promoter of hepatocytes exhibits high basal activity even in the absence of IL-6 induction and activation by the 3′ enhancer. In a pilot study by Ciliberto et al., a −1200/+44 bp region of hAAT promoter was reported to provide a high level of specific expression in hepatocytes [237]. Within this promoter various regions with different enhancer activity and specificity were determined based on the analysis of truncated variants (Figure 2B). The most proximal region, located at −137/−37, titled as a tissue-specific element (TSE), is capable of specifically enhancing the heterologous SV40 promoter 25-fold in hepatocytes. HNF4 and HNF1 binding sites have been identified in this region, and a mutation in one of them leads to a complete elimination of promoter activity [141,238]. Within the TSE, CRM8 (cis-acting regulatory modules) was later characterized, which has a high homology among many species and is essential for promoter function [241]. C/EBP, HNF1, HNF4, HNF6 and two HNF3 sites with different binding affinities were identified within −202/−70 region [242,243]. The intermediate region −261/−210 (DE1) is capable of enhancing the heterologous SV40 promoter in 40–50 times, which contrasts markedly with the fact that shortening the native promoter to −210 reduces activity only in 4–5 times. A core enhancer site was identified in this region, which is also found in many liver genes as well as viral enhancers. The site is presented in the 3′ enhancer and flanked by the AP1 and C/EBP sites [141,238,240]. The third major regulatory region DE2 of the promoter is located at −488/−356 and exhibits strong but non-specific enhancer activity [238].

Most further studies focused on the −721/+44 truncated promoter variant, since this promoter has an analogous activity to −1200/+44 promoter (Figure 2B) [237]. In one of the earliest studies focused on the comparison of promoter activity in primary hepatocytes the −732/+44 hAAT promoter demonstrated relatively weak activity compared to the 500 bp hAlb (human albumin) promoter and viral promoters [244]. However, in retroviral vectors the truncated −347/+56 hAAT promoter was superior to 820 bp mAlb both in vitro on differentiated hepatocytes and in vivo during hAAT expression in mice [245]. A similar result was obtained by comparison of the truncated −261/+44 hAAT promoter with −180/+16 mAlb where the hAAT promoter without enhancer showed higher activity, representing 40% of the CMV promoter activity. In the same study, the addition of 376 bp mAlb enhancer to the hAAT promoter increased the expression level of AAT from 20 to 70 ug/mL in mouse plasma (Figure 2C, Table 1) [141,246]. Therefore, both promoters found their clinical applications in which −261/+44 hAAT was used in AAV3B-mediated gene therapy VTX801 for Wilson’s disease, while EalbAAT (376 bp enhancer with −264/+20 hAAT) was used to treat acute intermittent porphyria (AIP) (rAAV2/5-PBGD) [151,154].

Nevertheless, VTX-801 gene therapy was terminated due to poor efficacy of the doses tested, despite promising results in preclinical studies [151]. Despite some improvement in patients’ quality of life, including a significant reduction in depression and anxiety, the rAAV2/5-PBGD therapy also proved to be not enough effective, as ALA (delta-aminolevulinic acid) and PBG (porphobilinogen) levels did not decrease even in the high-dose vector group with 1.8 × 10^13^ vg/kg [154]. To increase the therapy’s efficacy, two copies of the ADRES (ALAS Drug-Responsive Enhancing Sequence) enhancer were added to the EalbAAT promoter, that significantly improved the promoter’s activity in the presence of several porphyrinogenic stimuli. This modification of the promoter made it possible to achieve the same level of PBGD activity with 10 times lower viral load [247]. The same group of authors also obtained a hyperfunctional mutant PBGD variant that provided protection in AIP mice against PB-induced attack [248].

An interesting observation was that the addition of several copies of HNF3 binding sites does not enhance the hAAT promoter that is in contrast to mAlb. The previously characterized ApoE/HCR1 (hepatic control region 1) enhancer region was used to enhance the hAAT promoter. hAAT promoter and HCR1 enhancer combination subsequently became the most popular for providing liver-specific expression. The HCR1 region, which together with HCR2 control the expression of all E/C-I/C-IV/C-II cluster genes, was characterized into regulatory regions of varying lengths and enhancer activities [249,250]. The enhancer activity is driven by the presence of multiple binding sites for HNF3, HNF4, and C/EBP [251,252,253]. Originally, researchers used several copies of the 154 bp HCR1 enhancer region (PvuII-ApaI region) to enhance the −347/+56 hAAT promoter (Figure 2D). Interestingly, the greater promoter activity was attributed to the HCR1 enhancer in reverse orientation as the positioning of the enhancer downstream of the transgene coding sequence [47,254,255,256,257]. The use of a greater number of copies of the 154 bp (4 and 8) enhancer in the adenoviral (Ad) expression vector leads to alanine aminotransferase elevations and faster loss APOA1 transgene expression loss [255]. Nevertheless, immune tolerance to transgene was observed in mice using Ad and AAV vectors with the 4xApoE-hAAT promoter [258,259].

A key factor that advances the use of the hAAT promoter was the observation that the complete 711 bp HCR1 enhancer locus combined with 408 bp hAAT fragment resulted in a four-fold higher level of hFIX expression compared with four copies of the 154 bp enhancer [260]. Subsequently, the derived 1.1 kb HCR1-hAAT promoter was used in AAV and LV vectors in preclinical trials for the treatment of hemophilia B. It is important to note that liver-specific expression resulted in the immune tolerance induction in the animals [259,261,262,263]. However, the HCR1-hAAT promoter became most widespread as a variant in which the HCR1 enhancer region was reduced to ~320 bp containing full functional LCR activities and maintaining a similar promoter activity (Figure 2E) [260,264]. The promoter was at least twice more active than mTTR enhancer/promoter by normalized expression level [265]. Currently, the 730 bp HCR1-hAAT promoter is used for liver-specific expression in a various AAV-mediated gene therapies for the treatment of hemophilia B (Beqvez, idanacogene elaparvovec), Fabry disease (ST-920), Gaucher disease (FLT201, promoter titled as FRE76), Pompe disease (SPK-3006, vanglusagene ensiparvovec), Crigler–Najja syndrome (GNT003) and phenylketonuria (BMN 307; NGGT002) [157,162,165]. SPK-3006 gene therapy was terminated for strategic reasons while BMN 307 was discontinued based on preclinical results, where 6 out of 7 mice receiving the highest dose (2e14 vg/kg) developed tumors in liver necropsy with evidence for integration of portions of the AAV vector into the genome. This observation was most likely due to the extremely high dose of the vector [159].

The LSP1 (liver-specific promoter 1) promoter was obtained by the addition of another copy of the HCR1 enhancer, which enhances the HCR1-hAAT promoter by 2–3 times (Figure 2F) [20,266,267,268,269,270]. LSP1 promoter was used in preclinical studies of ornithine transcarbamylase (OTC) deficiency in mice and for the expression of piggyBac transposase for editing OTC-deficient patient-derived primary human hepatocytes [271,272,273]. However, LSP1 has not found clinical application despite its identical activity to TBG promoter [227].

Based on the capability of the AMBP (alpha-1-microglobulin/bikunin precursor) to enhance the hAAT promoter to the same degree as HCR1, two copies of the 160 bp AMBP enhancer were added to the 890 bp hAAT promoter to obtain the DC172 promoter, which has five times greater activity compared to previously reported DC190 (Figure 2G) [266,274,275]. An additional modification of DC172 involved inserting copies of the HCR1 enhancer; however, the promoter failed to be enhanced by the 154 bp HCR1 enhancer regardless of the copy number. This confirms the results of previous studies that AMBP and HCR1 enhancers do not have a combinatorial effect and are most effective when acting separately [256,266]. Copies of the 774 bp HCR1 enhancer can enhance the DC172 promoter by 1.5–2 times, but this significantly increases the size of the already long promoter, that limits its application [256]. The derived promoter was used in a more high-capacity Ad vector for regression and stabilization of advanced murine atherosclerotic lesions [276]. At the same time, the ~1.2 kb DC172 promoter was used for AAV-mediated expression of glucocerebrosidase in a mouse model of Gaucher disease, that results in the immune tolerance induction [275].

However, the great length of the promoters 730 bp HCR1-hAAT, ~1 kb LSP1, ~1.2 kbp DC172 limited their use in a number of gene therapies with larger CDS and for use in scAAV vectors. Based on analysis of TF binding sites, the HCR1-hAAT promoter was truncated to a 448 bp LP1 promoter during the development of the scAAV8 vector for hemophilia B therapy (Figure 2H) [174]. The shortened length of the promoter enabled its use in preclinical studies of AAV-mediated gene therapy for hemophilia A [277,278,279]. Some attempts were made to modify the LP1 promoter through nucleotide alteration to include HNF1 and HNF4 binding sites, but this provided a minor effect on promoter activity [280]. Due to the lower efficiency of assembling oversized AAV vectors such for hemophilia A gene therapy, further shortening of the promoter was required. Researchers from University College London obtained a 252 bp HLP (hybrid liver promoter) promoter during the development of hemophilia A gene therapy with a new FVIII-V3 variant which has increased expression efficacy (Figure 2I) [183]. HLP promoter design based on the results of the early pilot work mentioned above focusing on the characterization of the promoter functional regions [238]. It was reported that shortening hAAT promoter from −261 to −208 resulted in significant activity reduction, but no change was observed with further shortening to −137. Therefore, it was decided to combine the DE1 with TSE regulatory regions (Figure 2B) and add the first 34 bp of the 192 bp HCR1 enhancer used for LP1. The LP1 promoter was used in gene therapy for the treatment of hemophilia B (AMT-061, Hemgenix; scAAV2/8-LP1-hFIXco) and phenylketonuria (HMI-102 and HMI-103 are currently terminated), while HLP is used for the treatment of hemophilia A (BMN 270, Roctavian; GO-8) [171,176,177]. HMI-102 was discontinued due to the greater promise of HMI-103 therapy, designed to integrate PAH cDNA into the PAH locus via homology-directed repair (HDR). Despite encouraging preliminary results, HMI-103 was also terminated, presumably for economic reasons [281,282].

Despite reports of identical potency of LP1 and HLP promoters in AAV vectors in in vivo studies, in vitro studies on Huh7 cells demonstrated that the activity of various HCR1-hAAT promoter variants decreased with promoter length, and the HLP promoter provided the lowest FVIII expression [172]. The low activity of the HLP promoter is also confirmed in a large-scale study of enhancer and promoter combinations, where it is denoted as E01.A1AT [203]. To enhance HLP promoter the 34 bp HCR1 enhancer was replaced with 117 bp HCR1 [283]. The derived 335 bp in length HLP2 (FRE1) promoter was only 1.5 times less active than HCR1-hAAT (Figure 2J) [187]. HLP2 was used in gene therapies for Fabry disease (FLT190) and hemophilia B (FLT180a), which are currently terminated [186,188]. FLT180a was terminated after 10 patients had been enrolled because of changes to the clinical development plan and recruitment difficulties due to the COVID-19 pandemic (NCT03369444) while development of FLT190 in Fabry disease was paused to focus company resources on advancing FLT201 (NCT04040049).

HLP2 promoter underwent further modifications to provide fully identical to HCR1-hAAT activity. The 119 bp FRE72 promoter was obtained exclusively from the −130/+44 hAAT region by introduction of an internal deletion −50/+4 (Figure 2K). The obtained promoter had 1.5–2 times greater activity compared to its ancestor HLP2 promoter, despite the deletion of the most proximal regions involving the TATA box and TSS, which is quite unexpected in accordance with current concepts about the critical role of the basal promoter region. A similar approach with the proximal elements removal is observed for the new 139 bp Em-hAATsh promoter, which contains multiple deletions within the −155/+33 hAAT promoter region. In a similar manner the most proximal region was deleted which may indicate the low significance of the −40/+22 region of the basal promoter, additionally considering the extremely low activity of the −85/+9 hAAT promoter [284]. hAATsh consists of the following hAAT regions: −155/−141, −123/−106, −77/−41, +23/+33, and in combination with a synthetic enhancer, it results in a 10-fold activity increase compared to the HLP promoter (Figure 2L; Supplementary Material ZS802 Em-hAATsh) [190]. There were no reports yet on the use of the FRE72 promoter in clinical trials, however Em-hAATsh is presumably already utilized in the ZS802 hemophilia A gene therapy.

Thus, despite the long-standing combined use of the hAAT promoter and HCR1 enhancer, their application is becoming more widespread with an emphasis on reducing the promoter length. The 34 bp HCR1 enhancer is being investigated for the enhancing of other promoters, such as TBG and mTTR, while the AAT region is also used as an enhancer for the mTTR and Alb (*Xenopus laevis*) promoters [203,241]. The CRM8 region was further modified by aligning orthologs (CRMSBS2) or by returning 4 nucleotides to the pan-ortholog consensus HS-CRM8 sequence to the human consensus sequence within or proximal to the predicted transcription factor-binding sites [210,285].

The hAAT promoter offers a compelling example in which a truncated form outperforms the full-length form. The observed effect may be attributed to the presence of an upstream silencer and to differences in transcription factor-binding site density across promoter regions. A low number of such sites may indicate that the DNA remains in a nucleosome-associated, compact configuration, as transcription factors may be insufficient to displace the nucleosome [67,135].

## 6. Promoters Based on TTR Gene

Transthyretin, formerly known as prealbumin, is a highly conserved plasma transport protein expressed mainly in the liver, as well as in the choroidal plexus and retinal pigment epithelium. Notably, hepatic expression can be suppressed during the APR, since cytokines prevent hepatocyte TFs from binding to the promoter, whereas suppression does not occur in the choroidal plexus, indicating distinct regulatory mechanisms in these tissues [285,286].

**Figure 3:**
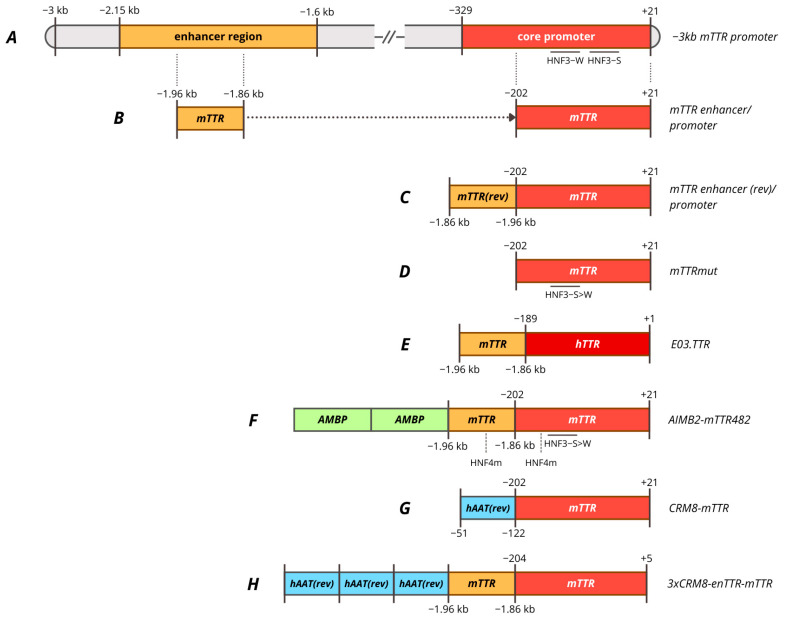
Promoters based on TTR gene. (**A**)—−3 kb murine TTR promoter regions. (**B**)—mTTR enhancer/promoter composed of mTTR promoter with 100 bp mTTR enhancer. (**C**)—mTTR enhancer (rev)/promoter (or enTTR-mTTR) composed of mTTR promoter with 100 bp mTTR enhancer in antisense orientation. (**D**)—mTTRmut, minimal mTTR promoter with HNF3 site mutated. (**E**)—E03.TTR composed of hTTR promoter with 100 bp mTTR. (**F**)—AIMB2-mTTR482 composed of mTTR promoter with HNF3 and HNF4 sites mutated, 100 bp mTTR enhancer with HNF4 site mutated and two copies of modified 162 bp AMBP enhancer. (**G**)—CRM8-mTTR composed of mTTR promoter with hAAT promoter in antisense orientation as enhancer. (**H**)—3xCRM8-enTTR-mTTR composed of mTTR promoter, mTTR enhancer and three copies of hAAT promoter in antisense orientation as enhancer. HNF3-W, HNF3 weak affinity binding site; HNF3-S, HNF3 strong affinity binding site; HNF3-S>W, substitution of HNF3-W site for HNF3-S site; HNF4m, modified HNF4 site for more affinity.

An early study focusing on the TTR promoter was conducted in 1986 by Costa et al. The researchers discovered an extremely high homology between the human and mouse *TTR* gene in the region 290n to the cap site after cDNA and the promoter region were sequenced. It was suggested that the promoter region necessary for TTR expression is located within 190 bp upstream of the cap site, since this region possesses the highest homology at 84%. Through deletion analysis, it was found that the NcoI-SstI enhancer element (−2150/−1600) is necessary for high liver-specific expression from the −329 promoter (Figure 3A) [287]. The activity of the −3000/−329 enhancer in antisense orientation was comparable to that in the native orientation when used to enhance both native and heterologous promoters. The same researchers succeeded in more accurately localizing the TTR enhancer region within −1.96/−1.86 kb (−1879/−1780 bp according to PubMed) and the core promoter region within −202 (Figure 3B). The identified enhancer region was able to enhance hepatocyte-specific expression from the heterologous β-globin promoter by 9-fold and contained 4 TF binding sites from hepatocyte nuclear extracts: two C/EBP, AP-1, and HNF4 sites. In addition, it was demonstrated that the mTTR core promoter and the mAAT enhancer region bind to similar TFs, such as HNF1, FOXA, C/EBP, and AP-1 [242,243]. Coordinated interaction between HNF1A, FOXA1/2, HNF4A, and HNF6A factors is necessary to provide core promoter activity, since mutation in a single site has a dramatic negative effect on promoter activity [285].

The derived ~320 bp mTTR promoter, consisting of a −202 promoter and a 100 bp enhancer, was compared with a −3 kbp promoter to endogenous expression upon the integration into the mouse genome. The 320 bp promoter provided full expression levels in the liver, but increased general expression in the brain outside the choroid plexus, while the −3 kb promoter provided low expression levels in the brain precisely restricting expression within the choroid plexus [288]. The 320 bp mTTR promoter was further compared with other liver-specific promoters in AAV vectors for factor IX expression including 190 bp mAlb, 730 bp HCR-hAAT, 845 bp LSP and 450 bp −219/+21 hFIX with the site 5 as an enhancer [289]. A scAAV vector with an mTTR promoter provided higher levels of FIX expression in vivo [265]. A similar enTTR-mTTR promoter, but with the mTTR enhancer in the antisense orientation, was used in gene therapies for the treatment of hemophilia A (TAK-754/BAX 888) and hemophilia B (AskBio009/BAX 335) (Figure 3C) [195,197]. BAX-335 was terminated due to rapid loss of transgene expression due to the use of a CpG-enriched sequence, which increases the immunogenicity of the vector and leads to the elimination of transduced cells, as it was also observed for DTX101 gene therapy [290]. However, a rapid decline in transgene expression within the first year was also observed for CpG-depleted TAK-754 gene therapy. The loss of transgene expression was not associated with liver dysfunction, and the cause remains unclear [291].

The 100 bp mTTR (antisense) enhancer was modified by random ligation of binding sites of various hepatocyte-specific transcription factors DPB, C/EBP, HNF1, HNF3, HNF4, and HNF6 to create the Enh1mTTR (ET) promoter [292]. ET promoter was used in lentiviral-based preclinical studies of hemophilia B therapy in mice and dogs, which resulted in stable but weak expression level of cFIX [293]. A shortened version of the promoter was subsequently used in the development of AAV5-mediated ANB-002 gene therapy for hemophilia B [200]. −202 core mTTR promoter was used in pilot studies of AAV-mediated gene therapy for hemophilia A, where the key requirement for the promoter was its minimal length with sufficient activity [287,294,295]. It was also found that the most important regions of the proximal mTTR promoter seemed to be the HNF4 and HNF3-S (strong affinity) binding sites, since mutations in these regions significantly reduced promoter activity even in the presence of an enhancer. The enhancer was able to compensate for mutations in the HNF1 and HNF3-W sites (weak affinity) sites. Furthermore, the conversion of the HNF3-W site to the HNF3-S site increased promoter activity 1.2-fold in the presence of a 100 bp enhancer and 1.8-fold in its absence (Figure 3D) [296]. The mTTR mut promoter with the mentioned mutation was subsequently used for factor VIII expression in AAV-mediated gene therapies SPK-8011 and NGGT003 for hemophilia A without an enhancer region due to AAV vector capacity limitations. This mutation was reported to increase FVIII expression in vivo in mice by 4-fold compared to the wild-type promoter [193]. Spark suspended phase 3 trials of SPK-8011 (NCT06297486) despite stable but low levels of FVIII activity (mild HA) over 4 years, which amounted to mean 7.4% [297]. The company stated its intention to use the enhanced function FVIII variant to improve the effectiveness of gene therapy. mTTR mut promoter was used in other preclinical studies of gene therapy for hemophilia A [298].

It should be noted that no distal enhancer element in human TTR similar to that in mice has been characterized, despite high homology in the proximal promoter region. The human TTR core promoter has poor activity without the 100 bp mTTR enhancer [299]. Various enhancers assessment was performed to increase activity that resulted in the generation of E03.TTR promoter, which represents a combination of the 100 bp mTTR enhancer with the 190 bp hTTR core promoter (Figure 3E) [203,300]. The E03.TTR promoter was used in gene therapies for hemophilia A DTX201 (BAY2599023) and Wilson’s disease UX701 [203,300]. DTX201 gene therapy provided promising results in clinical trials, achieving stable FVIII expression for at least 23 months despite the high content of CpG motifs [301].

Human and mouse TTR promoters were compared with 448 bp LP1 and 152 bp mFibr (modified human fibrinogen beta promoter) promoters in a factor VIII expression cassette. The LP1 and mTTR promoters, but not hTTR, provided the highest expression of factor VIII, that was confirmed by in vivo expression during hydrodynamic injection with plasmids. mTTR and LP1 promoters provided higher and more stable expression of FVIII in oversized vectors [302].

Due to its short length and compatibility with LP1 activity, mTTR was modified for use in gene therapies with large coding sequences. Nambiar et al. created the mTTR202opt promoter by replacing the HNF3 site with a variant with more affinity, as used for SPK-8011 and NGGT003, and replacing the HNF4 binding site in the proximal region of the promoter. Next, a modified mTTR enhancer was added to the resulting promoter, in which the HNF4 binding site was replaced by analogy with the proximal promoter, resulting in the mTTR482 promoter. mTTR202opt and mTTR482 provided more than twice the expression of FVIII in vivo compared to the WT-202 mTTR promoter [207]. Two copies of the 162 bp modified AMBP enhancer (to make it more affine to HNF3 and HNF4 factors) were added to mTTR482 to obtain the mAlMB2-mTTR482 promoter, which was used in SAR444836 gene therapy for phenylketonuria [207] (Figure 3F). Interestingly, the combination of the TTR promoter with the truncated AMBP enhancer led to the formation of FVIII-specific neutralization antibodies, that may raise some concerns in a number of therapies with high immunogenicity of the secreted transgene [203].

Several approaches were developed to use other genes as enhancers to boost the mTTR promoter. It was demonstrated that the proximal region −202/−70 hAAT can act as an enhancer for the heterologous βb-globin promoter, which could enhance transcription more than the 100 bp mTTR enhancer. In the study by Chuah et al., researchers used computational methods to search for highly conserved cis-acting regulatory modules (CRMs) in highly expressed liver genes. The regions they found were used as enhancers for the mouse TTR promoter, including the hTTR promoter region denoted as CRM10 [241]. The 71 bp CRM8, which is a part of the previously characterized tissue-specific element of the hAAT promoter region, was able to enhance the mTTR promoter 7–10 times and provided liver-specific expression in non-human primates delivered by the scAAV9 vector (Figure 3G) [141,241]. As a result of further modification, the hsCRM8-mTTR promoter, also known as HSh-TTR or HHS4-TTR, proved to be 2–3 times more active than the modified LP1 in vivo [280]. The Hsh-TTR promoter was used in preclinical studies of gene therapy for hemophilia B in non-human primates, as well as in the study of various FIX variants obtained through ancestral sequence reconstruction [303,304,305].

Independently of this modification the CRMSBS2-TTRm promoter was obtained by aligning the *SERPINA1* gene sequence (using ENCODE Alignment) and selecting low-conservative variants. The promoter is used in clinical trials of SB-525 (giroctocogene fitelparvovec) gene therapy for hemophilia A [209,211]. Interestingly, up to half of patients experienced supraphysiological FVIII activity levels, requiring anticoagulant therapy until physiological activity levels were restored. This may be related to both high promoter activity and high vector doses [306].

The use of a larger number of copies of CRM8 can significantly enhance the mTTR promoter, but presence of an mTTR enhancer is still necessary to provide maximum TTR promoter activity, that is an obstacle to its use in gene therapies with a large transgene coding sequence, as in the case of hemophilia A [196,307]. Three copies of an unmodified CRM8 fragment were used to enhance the enTTR-mTTR promoter, as in the case of VGB-R04 gene therapy for hemophilia B [213]. However, TAK-748 therapy utilizing the same promoter was terminated prior to the start of trials due to expectations of low therapeutic efficacy [196,212].

## 7. Promoters Based on Albumin (Alb) Gene

Albumin is the most abundant plasma protein (40–60% of total protein, 35–52 g/L in human plasma), synthesized exclusively by hepatocytes. Among its principal functions, albumin maintains blood oncotic pressure and transports various ligands, including fatty acids and hormones [308,309]. The exceptionally high level of liver-specific albumin expression—accounting for up to 15% of total hepatic protein synthesis—has prompted extensive investigation into its promoter as a powerful tool for achieving tissue-specific gene delivery in the context of gene therapy [310,311]. Building on the robust endogenous synthesis of albumin, various strategies involving zinc-finger nuclease (ZFN)-mediated integration of therapeutic transgenes directly into the albumin intron were pursued (SB-913, SB-318, SB-FIX). Nevertheless, these strategies demonstrated insufficient clinical efficacy in vivo, leading to a reorientation of research approaches toward the use of transcriptional regulation via the albumin promoter (without genomic integration) in AAV-mediated gene therapy systems [312,313,314].

**Figure 4:**
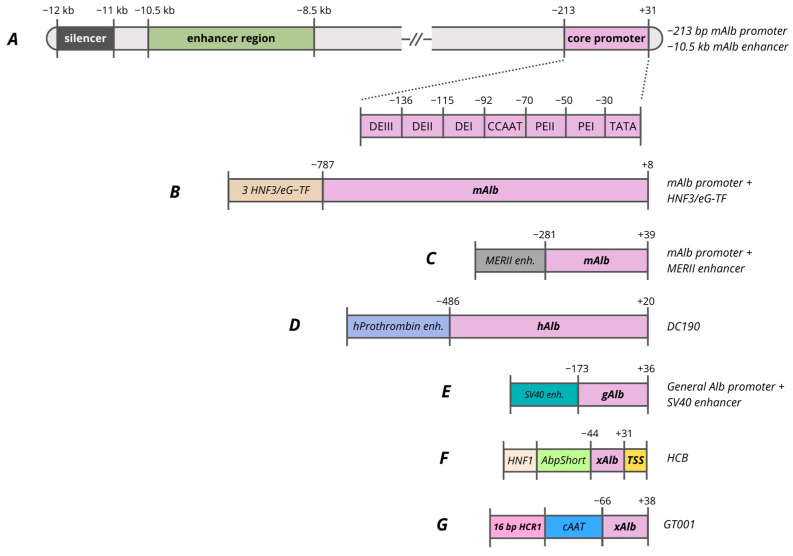
Promoters based on Alb gene. (**A**)—−213 bp albumin promoter and −10.5 kb enhancer regions. (**B**)—mAlb promoter composed of mAlb promoter with 3 copies of HNF3/eG-TF. (**C**)—mAlb promoter composed of mAlb promoter with MERII enhancer. (**D**)—DC190 promoter composed of hAlb promoter with hProthrombin enhancer. (**E**)—gAlb promoter composed of general Alb promoter with SV40 enhancer. (**F**)—HCB promoter composed of xAlb promoter with consensus TSS, AbpShort and HNF1. (**G**)—GT001 vector composed of xAlb promoter, cAAT and 16 bp HCR1.

Pilot studies suggested that the region of the albumin promoter (PAlb) lies between −31 and −213 and contains six potential cis-acting elements, divided into distal (DE) and proximal (PE) [309,315,316]. The regulatory regions of the promoter include the TATA box, CCAAT box, PEI, PEII, DEI, DEII, and DEIII (Figure 4A). The TFs responsible for transcriptional activity of the albumin promoter include HNF1, C/EBP, DBP and nuclear factors Y and 1 (NF-Y and NF-1). It has been established that promoter regions are highly conserved in rat, mouse, and human *albumin* genes. The characteristic pattern of DNA–protein interactions observed in these promoters is preserved throughout evolution, reflecting the presence of hepatocyte-specific transcription factors that have been conserved over time [317].

Considering the functional significance of transcription factor-binding sites, it is noted that deletion of the HNF1 site completely blocks both promoter and enhancer activity [318]. Originally, this proximal region was identified as a negative regulatory element (NRE), owing to the presence of a GATC motif susceptible to methylation. Studies employing methylatable bacterial strains for plasmid preparation revealed a threefold reduction in transcription, underscoring the pivotal roles of HNF1 and epigenetic regulation [315]. The NF-Y site mutation reduces promoter activity by half, but distal enhancers can partially compensate for this effect. The C/EBP(GCAA) site mutation at position −124 leads to a 15-fold reduction in transcription, demonstrating the key function of C/EBP factors.

Analysis of 5′-deletions in the rat albumin promoter has revealed that the size of the promoter region is crucial for its function. A minimal construct extending to −84 bp, with or without a 2 kb albumin enhancer and containing the HNF1 and TATA box, exhibits only low-level activity. For full tissue specificity and proper responsiveness to enhancers, a promoter region extending to −170/−175 bp is required [318,319]. Comparison of variants of the human albumin promoter in reporter adenoviral vectors identified fragment −173/+36 as the most active, demonstrating high promoter activity in Hepa1–6 mouse liver cells (Figure 4E). The −247/+36 variant showed the lowest activity, indicating the presence of negative regulatory elements in the −247/−173 or unfavorable interaction with the SV40 enhancer used in the study [310].

Several studies reported that the albumin promoter, when paired with its native enhancer, yields only modest levels of expression in vivo [141,320]. However, when regulatory elements from the albumin promoter, including modified variants, are combined with enhancer and promoter regions derived from other highly expressed liver genes, the resulting constructs achieve substantially higher levels of transgene expression.

The albumin enhancer (EAlb), located between −8.5 and −10.5 kb upstream of the transcription start site, harbors functional domains—eH-TF, C/EBP, HNF3, and NF-1—that serve as binding sites for liver-specific transcription factors, including HNF4A, C/EBPα, and FOXA2 [321,322,323]. This enhancer regulates the promoter by interacting with proximal elements: HNF1 directly activates the promoter, while architectural factors such as HMG-I(Y) and NF-Y fine-tune activation through chromatin looping, thereby ensuring robust and stable transgene expression in the liver [318,324,325].

It was identified that the region located further upstream, −12/−11 kb, is a silencer, as it inhibits transcription in the case of both the homologous albumin promoter and the heterologous herpes simplex virus thymidine kinase promoter (HSV-TK promoter) [326]. In the region −10,284/−9904 bp, point mutations in the C/EBP, C/EBP-RF, HNF3, and eH-TF sites impede enhancer function, and deletion of the eH-TF site completely negates activity, indicating the need for cooperative interaction of all these factors for maximum liver-specific expression [326]. Experiments demonstrated that the albumin enhancer Ealb (−10,500/−8500 bp, 370 bp) has limited effectiveness on its own and only functions with robust support, which was employed to construct the EalbAAT promoter mentioned above (Figure 2C) [141,154]. Understanding the critical role of various regulatory elements has allowed researchers to create optimized promoter constructs for the expression of reporter systems and transgenes.

In several studies the modified mouse albumin promoter (−787/+8 bp) was enhanced by three copies of HNF3/eG-TF binding sites its enhancer region (Figure 4B) [327,328]. This ensures high expression of biologically active human factor VIII/hAAT in HepG2 cells and animals, using retroviral and adenoviral vectors [245,329,330,331].

The minimal mouse albumin promoter (−281/+39 bp), coupled with the α-fetoprotein enhancer (MERII) (Figure 4C), the 5′ intron of factor IX, and the 3′ intron of albumin (3′iALB), achieves stable secretion of alkaline phosphatase in mouse liver, matching the CMV promoter’s efficiency for a full year. The inclusion of 3′iALB after the coding region increases expression fivefold compared to variants without it, whereas the distal albumin enhancer in the construct (−10,284/−9904 bp) does not provide long-term activity [320]. The recombinant human liver-specific DC190 promoter contains the human albumin promoter (−486/+20 bp). To enhance transcriptional activity, two tandem copies of the human prothrombin enhancer (−940/−860 bp), a hybrid intron (three-way adenovirus donor leader/mouse immunoglobulin acceptor) and a BGH polyA signaling sequence for transcript stabilization (Figure 4D) [256,332,333,334]. Using the DC190 promoter in the AAV2/DC190-αgal construct, the expression of α-galactosidase A in mouse livers increased by 15-fold compared to the CMV promoter [335]. The addition of the hAAT intron further increased transgene expression due to splicing optimization and mRNA stabilization.

The promoter of the frog (*Xenopus laevis*) *albumin* gene, containing a 13-nucleotide HP1 element (CNXNNTTINNNNNC) and a TATA box, was used as a minimal liver-specific promoter capable of sustaining expression in hepatocyte cells in vitro. At the same time, proximal NF-Y/CCAAT, HNF1 and other sites are not essential components of the construct. Similar HP1 motifs have been found in the promoters of mouse and human *albumin*, *α-fetoprotein*, *fibrinogen beta chain*, and *α*_1_-*antitrypsin* genes, demonstrating significant evolutionary stability of this element [336]. The frog Alb promoter was used in the clinical trial of the gene therapy drug for hemophilia A–ASC618 (NCT04676048) as part of the minimal synthetic promoter HCB (HNF1-AbpShort-SynO-TSS, 146 bp). It consists of SynO (41 bp) from the 5′ UTR of X. laevis albumin as the core promoter, AbpShort (61 bp), a truncated α-microglobulin enhancer, a consensus TSS (Figure 4F) and provides 14 times greater FVIII activity compared to the clinically used HLP promoter (252 bp) in the AAV-FVIII [214]. Similarly, in the hemophilia A therapy (GS1191-0445) using the AAV8 vector, a modified frog Alb promoter (−66/+38, xAlb) was combined with elements from dog AAT (cAAT) and human AAT (hAAT), along with a synthetic enhancer (Figure 4G) [218].

## 8. Promoters Based on AFP Gene

Alpha-fetoprotein (AFP) is a serum glycoprotein, a member of a multigene family that also includes the albumin gene, which is closely linked to it [337]. During embryonic development, AFP is actively expressed in the visceral endoderm of the yolk sac, the fetal liver and the gastrointestinal tract, but after birth, AFP expression in the liver decreases sharply to practically undetectable levels—the concentration of AFP mRNA decreases by 10^4^ times 3–4 weeks after birth [324,338,339]. Paradoxically, this limitation makes AFP a perfect tool for cancer-selective therapy. In hepatocellular carcinoma, AFP is reactivated in about 70–80% of cases. This creates a unique opportunity to target tumor cells specifically, without harming healthy liver cells [340,341,342]. For liver-specific delivery to healthy hepatocytes, hybrid constructs are developed. These combine AFP enhancers with constitutively active liver-specific promoters, allowing the powerful enhancer properties of AFP elements to be utilized while maintaining the ability to express in healthy hepatocytes.

The AFP promoter region can be divided into two main classes of regulatory elements: the proximal tissue-specific region (−85/−52 bp from the transcription start site) and distal enhancers (−7.6/−1.0 kb), both of which are critical for transcription and tissue-specific expression of AFP. The enhancer domain is represented by three functionally independent elements (I: −1.0 to −3.8 kb; II: −3.8 to −5.3 kb; III: −5.3 to −7.6 kb), which have significant functional redundancy and function independently of their position and orientation relative to the promoter. The most active region is the most proximal element I (−3.8/−1.0 kb), which enhances transcription from the HSV TK promoter by 50-fold [343]. In addition to the activating elements, two repressor sites (−1822/−951 and −402/−169 bp) have been identified, the former being more active and capable of blocking the action of enhancers [344]. Distal enhancers need a PCE (promoter-coupling element) to function properly. This element activates HNF1-binding sites and ensures that distant enhancers interact with proximal promoter elements [318]. AFP regulation is characterized by the involvement of classical liver-specific transcription factors, including HNF1, C/EBP and other hepatocyte nuclear factors [214,243]. The minimal proximal promoter AFP mouse (61 bp) was tested in a study to develop a promoter for hemophilia A gene therapy. In a construct containing AbpShort-HP1-AFP-TSS, the minimal promoter demonstrates a significant increase in expression in vitro [214].

## 9. Promoters Based on TBG Gene

Thyroxine-binding globulin (TBG) is a liver-derived glycoprotein responsible for the transport of thyroid hormones in blood serum. Since TBG is predominantly synthesized in hepatocytes, its promoter has become a key tool for achieving tissue specificity in gene therapy for inherited liver diseases. Minimal promoter activity is observed in non-target tissues, including the spleen, kidneys, and large intestine [149].

The structural and functional organization of the TBG promoter has been analyzed since 1993 [345]. The key HNF1 site at positions −77/−65 bp is the most critical, as its mutation completely eliminates promoter activity. Additionally, there are a TATA box (+26 bp), C/EBP, an NF-1 site (−175 bp), and several AP-1 and HNF3 sites associated with tissue specificity. TBG promoter variants with different lengths were characterized in order to discover the most active variant. The study tested three additional TBG promoter fragments TBG1 (−253/−26 bp), TBG2 (−635/−26 bp) and TBG3 (−1190/−26 bp) and compared them with the original −435/−26 bp fragment. The shortest fragment, TBG1, showed a strong drop in activity, while extending the promoter to −635 bp slightly increased expression; however, further extension to −1190 bp reduced activity again. The −435/−26 bp fragment proved to be the most effective and practical, providing high transgene expression without unnecessarily increasing construct size [149,345,346].

Based on TBG, the liver-specific promoter (LSP) has been created, combining the −382/+3 bp fragment of TBG, two copies of the AMBP enhancer (α1-microglobulin/bikunin, −2804/−2704) and the 71 bp leader sequence [347]. LSP is actively used in AAV vectors for the treatment of hemophilia A and B. In animal model experiments, it provides stable expression of coagulation factors and correction of the disease [51,256,277,348,349,350]. A dual-vector system has been created to treat hemophilia A. It separately expresses the heavy and light chains of FVIII using vectors controlled by the LSP (659 bp). This system demonstrates equivalent efficacy to the single-vector approach using a short synthetic 368 bp IGBP (insulin-like growth factor-binding protein) promoter [351].

In clinical studies, AAV8.TBG.hARSB therapy with two copies of 101 bp of the AMBP enhancer and the −474/+3 region of the TBG promoter maintained 38–67% of normal arylsulfatase B (ARSB) activity in patients with mucopolysaccharidosis type VI (MPS VI) during 45 months of follow-up [219]. In the treatment of Pompe disease, ACTUS-101 gene therapy utilized LSP to express α-glucosidase. Safety and sustained increase in GAA levels in patients in phase 1/2 was demonstrated [222]. Hemophilia B (DTX101) therapy with LSP in AAVrh10 reduces the annual bleeding rate by 71% and provides sustained factor IX activity > 5% in 82% of patients after 24 months [221]. However, the primary endpoint of the clinical trials in terms of IX activity level was not achieved, and the therapy was discontinued.

TBG promoter with AMBP enhancer (680 bp) and Kozak-like sequence in the construct for the treatment of ornithine transcarbamylase deficiency (OTCD) (scAAV8.TBG.hOTCco) provides 60% of normal OTC activity on day 28 [225].

## 10. Synthetic Promoters

Over recent decades, the rapid development of computational approaches analyzing regulatory element conservation and multi-omics data has enabled rational design of fully synthetic regulatory elements. Omics datasets (particularly ChIP-seq for profiling histone modifications and TFBS, ATAC-seq for assessing chromatin accessibility, as well as spatial and single-cell transcriptomics) provide valuable insights into regulatory grammar and the development of synthetic promoters. These datasets allow the identification of natural regulatory elements, evaluation of their activity, and subsequent application to bioinformatic tasks such as classifying sequences as promoter or non-promoter, predicting promoter activity, and generating de novo promoters.

Promoter design typically involves an iterative process of sequence generation, activity prediction, and subsequent optimization. Various deep learning approaches, such as convolutional and recurrent neural networks, are used to identify and enrich synthetic constructs with motifs specific to the target tissue. The current state of these approaches is reviewed by Wang et al.; in this work we focus on tools and synthetic constructs for liver-directed applications [352].

For enhancer identification, a hybrid neural network, Enhancer-CRNN was developed [353]. Trained on publicly available histone modification data, it accurately localizes natural enhancers, including those in the HepG2 cell line.

DeepLiver, a hierarchical convolutional neural network, was trained to predict enhancer activity and hepatocyte zonation using multi-omics data [354]. The model identified key transcription factor motifs, such as HNF4A, C/EBPa, HNF1A, FOXA1, and AP-1, as drivers of enhancer activity, and its predictions strongly correlated with experimental MPRA data, including for synthetic sequences.

The CODA platform generates 200-bp synthetic enhancers for three cell lines (HepG2, K562, and SK-N-SH) based on activity predictions from the Malinois convolutional neural network, followed by sequence optimization [355]. Analysis of the optimized sequences showed enrichment of binding sites characteristic for the target cell types; for HepG2, these included TFBS for HNF1B and HNF4A. Subsequent testing in transgenic zebrafish demonstrated liver expression in 27 out of 36 cases.

Regarding the practical application of synthetic constructs, the Lxp2.1 promoter was designed to ensure selective transcriptional activity in hepatocytes and comprises a minimal promoter supplemented with two enhancer elements. Its structure contains three binding sites for HNF1, two sites each for HNF3 and HNF4A, and one site for SP1. The Lxp2.1 promoter is part of the vector systems BBM-H901, the first gene therapy for hemophilia B approved in China for clinical use, and BBM-H803, a gene therapy for hemophilia A [229]. Notably, during therapeutic development, the arrangement and relative spacing of binding sites have been shown to play a major role in the efficiency of synthetic constructs.

Another example of a synthetic construct is the 54-bp Em enhancer, incorporated in a construct developed for ZS802 hemophilia A gene therapy [189]. This enhancer was used in combination with the human AAT core promoter and contains HNF4A, C/EBPa/b, FOXA2, HNF1A/B, and D site-binding protein (DBP). The AAT core promoter was also used in G6PC_COMP_v1 and G6PC_COMP_v3. The synthetic sequences contain TFBS, which increase expression in hepatocytes up to 20-fold over the LP-1 promoter [172]. The TFBS are organized as consecutive pairs: HNF1/HNF3, HNF3/HNF3, and C/EBP/HNF4, separated by spacers.

Although we cannot precisely determine how the promoters described above were rationally designed, fully synthetic promoters nonetheless constitute an important tool. Their further development may be more effectively aided by design methods based on neural networks.

## 11. Conclusions and Future Prospects

The importance of the liver as a central metabolic organ cannot be overstated. The large number of secreted proteins, the maintenance of balance between carbohydrate and lipid metabolism, and its special immune status make the liver an attractive target for gene therapy for various diseases. Current strategies for developing promoters for gene therapy build on fundamental principles of the liver’s regulatory network. More specifically, these strategies are based on the interaction of transcription factors with natural promoters of tissue-specific and highly expressed genes. Despite significant progress in identifying many specific regulatory elements, our current knowledge does not allow us to precisely and flexibly regulate expression, which is necessary for a number of metabolic diseases. Furthermore, researchers face several challenges in developing liver-directed gene therapies due to inherent limitations of delivery vectors.

Early attempts at liver-directed gene therapy faced significant hurdles related to promoter choice. The use of adenoviral vectors was complicated by the rapid loss of transgene expression when driven by strong ubiquitous promoters, including viral ones [356,357]. Although the use of liver-specific promoters was a shift to reduce the overall immunogenicity of the vector resulted in a more stable and prolonged therapeutic levels of transgene expression, these promoters failed to eliminate the immunogenicity of the adenoviral vector itself, and loss of transgene expression persisted [258,358,359,360,361,362]. Similarly, initial studies using AAV vectors with viral promoters also failed to achieve stable, high-level transgene expression in target tissues like the liver and muscle [33,143,363,364]. A critical turning point was the discovery that ubiquitous promoters increases the risk of inhibitor formation against the expressed transgene, whereas liver-specific promoters facilitate the immune tolerance [259,365]. In 1999 a seminal research study demonstrated the use of a liver-specific LSP in an AAV vector, achieving stable FIX expression in mice at levels up to 10 µg/mL—twice the normal human physiological concentration [347]. Subsequent studies showed that liver-specific promoter expression could induce transgene-specific immune tolerance, underscoring the safety and efficacy of AAV vectors in gene therapy [259,275,366]. Transgene expression in the liver is actively applied in therapies in which the therapeutic protein needs to perform its physiological effect in muscle cells, such as in Pompe disease. Directing expression to the liver serves to induce immune tolerance, since the use of muscle-specific or ubiquitous promoters leads to the formation of transgene-specific neutralizing antibodies, as observed in clinical trials of AAV1-CMV-GAA [367,368,369].

To enable dual expression in the liver and muscles, new promoters such as LiMP (liver–muscle promoter) have been developed. LiMP combines the previously characterized strong liver-specific HCR1-hAAT promoter with the spC5.12 promoter, which is active in skeletal and cardiac muscle. The promoter was utilized in preclinical trials for the treatment of Pompe disease and MPS IVA (mucopolysaccharidosis type IVA) [370,371,372]. To date, all approved liver-directed gene therapies are based exclusively on the delivery of AAV vectors. mRNA delivery via lipid nanoparticles (LNPs) is also being actively developed and is currently in clinical trials However, mRNA delivery cannot provide sustained expression and requires regular drug administration [373].

An additional advantage of using liver-specific promoters in AAV vectors is their potency to induce immune tolerance in subjects with pre-existing antibodies, which may be highly relevant in human therapy [51,366,374]. Currently, the presence of neutralizing antibodies to the transgene in patients is the exclusion criteria for gene therapy. However, there are encouraging results suggesting the possibility of eliminating pre-existing neutralizing antibodies with appearance of the transgene therapeutic activity in the same time in humans, as recently demonstrated in clinical trials of BMN 270 gene therapy (NCT04684940) [53]. All these results highlight the critical role of liver-specific promoters in ensuring the efficacy and safety of gene therapy.

Despite greatest frequency of liver-specific promoters to use in tissue-specific gene therapies and the diversity of their variants, the most of liver-specific promoters are based on just three genes including *AAT*, *TTR*, and *TBG* [140]. Up to half of the gene therapies presented in this review utilize promoters based on *AAT* (48.7%), 20.5% on *TTR*, 7.7% on combinations of *AAT* and *TTR*, and 10.3% on *TBG* (Table 1). It should be noted that all three AAV-mediated liver-directed FDA approved gene therapies use liver-specific promoters based on human AAT promoter with ApoE/HCR1 enhancer, including Hemgenix, Beqvez, and Roctavian. Although these promoters proved their effectiveness, new promoter variants based on other genes and/or computational technologies need to be developed to improve the efficacy of gene therapies.

In summary, our data indicate that a universal strategy for designing liver-specific promoters is to adopt the architecture of highly expressed hepatic genes and to reduce it in a rational way. In most cases, such promoters are constructed by truncating natural sequences to minimal functional regions, by varying their length, and by testing expression constructs in vitro [375]. TF footprinting is an informative tool for rational truncation. Three widely used promoters (AAT, TTR, TBG) contain TFBS that are strongly associated with high expression in vivo and in vitro: HNF1, FOX, CEBP, MyoD, LEF-1, LEF-1/TCFβ, and Tal1β/E47 for AAT; HNF1, CEBP, FOX, LEF-1, LEF-1/TCF, and MyoD for TTR [241]; and HNF1 and HNF3 for TBG [345]. Preference is also given to promoters of genes that show more predictable and stable expression. For example, the ALB promoter has not been widely used, apparently because of its more complex regulatory organization and its dependence on the physiological state of the liver parenchyma, including changes during inflammation and nutritional stress [376,377].

Unfortunately, despite numerous studies investigating or creating promoters based on the *hAAT*, *TTR*, and *TBG* genes, there is no information on a direct comparison of all the promoters mentioned in this review within a single study. Comparing promoter activity based on multiple studies is complicated by different study design approaches. First, various reference promoters can be used to evaluate relative promoter activity, which prevents general normalization to one specific promoter. Second, promoter activity can be assessed both at the level of transgene mRNA and by the amount of reporter or therapeutic gene protein. Third, various approaches can be used for transgene expression, including both plasmid expression vectors and viral vectors, that also impacts expression efficiency. Fourth, and most importantly, the activity of different promoters can be assessed in vitro in various cell lines and in vivo in animal models. All these variables prevent direct comparison of both the activity and safety of promoters based on the results of different studies. However, with the development of sequencing technology, high-throughput methods for screening promoters have become available, such as based on the creation of barcoded libraries, that allow the activity of multiple promoter and enhancer elements to be evaluated in parallel [150,378,379,380].

It was recently reported that the 2.2 kb GFAP (glial fibrillary acidic protein) promoter provides the highest level of expression in the liver among a variety of strong ubiquitous and liver-specific promoters, that was rather unexpected, since GFAP promoter is known for astrocyte-specific expression in the central nervous system. An apparent limitation of the GFAP promoter is a large length, and the currently existing truncated variants do not provide the same activity [150,381]. It is also important to mention the previously described promoters that have not yet found application in gene therapies, such as the ADH6 (class V alcohol dehydrogenase) promoter, which has comparable activity to hAAT [266,382]; the APOA1 promoter used in Ad vectors [254,358]; the APOC2 promoter, which is more than 6 times more active than APOA1 [255,383]; the IGBP promoter, which in combination with 2 copies of the AMBP enhancer provide similar to the HCR1-hAAT promoter activity [277]; the AHSG (or pp63 for rat, alpha-2-HS-glycoprotein) promoter, which provide greater activity compared to the β actin promoter [384]; the L-PK (liver-type pyruvate kinase) promoter, which has activity similar to that of the mAlb promoter despite its short length [385]; the ALDOB (aldolase B) promoter, which can be enhanced more than 100-fold by enhancer regions within the intron [386]. In the future these promoters after optimization may find an application in liver-directed gene therapy. However, for some promoters, a serious limitation for application is the inducibility from external signals, such as cytokines IL-1 and IL-6 (acute-phase proteins) or hormones. The promoters of *HP* (haptoglobin) [387], *Hpx* (hemopexin) [388], *SERPINA3c* [389], *FGA* (fibrinogen alpha chain) [390,391], *FGB* (fibrinogen beta chain) [392], *PEPCK* (phosphoenolpyruvate carboxykinase) [244,245], *L-PK* [385], and *CRP* (C-reactive protein) [393] genes have weak basal activity without induction, but at the same time can be extremely active in vivo depending on the body conditions, that makes them difficult to control and unsafe for in vivo application. A promising solution to this problem could be the modification/replacement of inducible regulatory sites and maintenance of always-on high promoter activity without regulation by external signals. However, in practice such modifications often diminish not only inducible activity but also basal promoter activity [387,391,392].

The use of strong liver-specific promoters can overcome barriers to the effective and safe application of liver-directed gene therapies through increased transgene expression and reduced viral load at the same time. The ideal promoter for gene therapy (not only for liver-directed) should provide stable high tissue-specific expression levels with its minimal size. However, in practical application, the choice of promoter is based on the characteristics of the delivery vector. The use of scAAV or transgenes with a large CDS greatly restricts promoter length, which can negatively impact its activity. Moreover, recent studies suggest that the use of strong promoters may be undesirable, despite their specificity. The utilization of a stronger promoter with a reduced vector dose results in a greater metabolic load within a smaller number of transduced cells, which also negatively affects expression stability. The strongest promoters can lead to unfolded protein response (UPR), endoplasmic reticulum (ER) stress, loss of transduced cells, and the formation of neutralizing antibodies to the transgene [34,203,394].

However, it is important to note that these studies were conducted in the context of preclinical studies of AAV-mediated gene therapies for hemophilia A for endogenous expression of factor VIII, which has low secretion efficiency and ability to induce UPR and ER stress [395]. The results may also be influenced by the use of oversized vectors, the AAV manufacturing platform, and the vector serotype [396]. This suggests that the immunogenicity of promoters should primarily be evaluated in the context of the characteristics of the vector used, the expressed transgene, and the route of vector administration. Thus, tightly regulated promoters of special interest could find wide application in the development of gene therapies for metabolic diseases. However, to date, approaches to promoter selection for the treatment of metabolic disorders or coagulation factor deficiencies do not differ, focusing on stable high transgene expression.

A particularly attractive prospect for highly effective gene therapy is the ability to regulate promoter activity in a controlled manner via internal stimuli—for example, to stimulate insulin expression in response to elevated glucose levels or to reduce transgene expression at supraphysiological levels [397]. Such features could significantly expand the list of diseases suitable for gene therapy and increase safety. The use of synthetic promoters remains limited at the current stage of genetic engineering development, although several clinical applications have been presented in this review. The methods for generation of these promoters are not always clearly established, but it can be concluded that they represent a combination of well-known liver-specific TFBS. The most effective sequences and optimal distances between them were experimentally determined for these sites. Attempts to create synthetic promoters using deep learning technologies experience a number of challenges. The key challenge is developing models that can accurately predict the correlations between sequence features and promoter activity. The primary complexity is accounting for spatial regulation, DNA structure and plasticity, and interactions with regulatory proteins. Large amounts of specialized annotated genomic data are required to identify patterns and model the complex spatial organization of regulatory elements. One challenge is the necessity to develop models that can accurately predict the relationships between sequences and their activity levels. The principal difficulty lies in accounting for spatial regulation, DNA structure and plasticity, and interactions with regulatory proteins. Large amounts of specialized annotated genomic data are required to identify patterns and model the complex spatial organization of regulatory elements. In addition, the accumulation of MPRA data is particularly important for model training, as it provides predictive power in the context of synthetic sequences. Training exclusively on natural sequences faces the problem of limited data volume, which can be partially addressed through data augmentation methods; however, their use may introduce distortions. Future research should focus on developing new architectural solutions aimed not only at identifying specific sites, but also at accounting for their interactions and generating diverse candidate sequences.

In summary, further development in the tissue-specific promoters area requires a comprehensive multidisciplinary approach, involving fundamental research to a deeper understanding of gene expression regulation, including epigenetic mechanisms. This also suggests the need to study aspects of intrahepatic signaling, which plays a key role in inducing cellular response and adaptation. In addition, a critical objective is to create and improve computational architectures capable of effectively extracting and processing information about complex epigenetic and signaling features. The accumulation of relevant data will serve as the platform for developing more accurate models to predict activity and generate synthetic constructs implemented in practical use.

## Figures and Tables

**Table 1:** Liver-specific promoters utilized in gene therapy.

Promoter	Gene of Origin	Length, bp	Features	Disease	Gene Therapy	Ref.
hAAT	Human *AAT*	305	−264/+41 hAAT promoter	Wilson disease	VTX801	[151,152,153]
EalbAAT	Human *AAT*, murine *Alb*	673	−264/+20 hAAT promoter with 376 bp mAlb enhancer	AIP (acute intermittent porphyria)	rAAV2/5-PBGD	[154,155,156]
ApoE/HCR1-hAAT	Human *ApoE/HCR1* and *AAT*	727	−355/+42 hAAT promoter with 321 bp ApoE/HCR1 enhancer	Hemophilia B	Beqvez (fidanacogene elaparvovec, SPK-9001)	[157,158]
ApoE/HCR1-hAAT	Human *ApoE/HCR1* and *AAT*	732	−353/+50 hAAT promoter with 321 bp ApoE/HCR1 enhancer	Phenylketonuria (PKU)	BMN 307	[159,160]
ApoE/HCR1-hAAT	Human *ApoE/HCR1* and *AAT*	725	−355/+43 hAAT promoter with 321 bp ApoE/HCR1 enhancer	Phenylketonuria (PKU)	NGGT002	[161]
ApoE/HCR1-hAAT	Human *ApoE/HCR1* and *AAT*	723	−355/+38 hAAT promoter with 321 bp ApoE/HCR1 enhancer	Fabry Disease	ST-920	[162,163,164]
ApoE/HCR1-hAAT	Human *ApoE/HCR1* and *AAT*	727	−355/+42 hAAT promoter with 321 bp ApoE/HCR1 enhancer	Crigler-Najjar syndrome	GNT0003	[165,166,167]
ApoE/HCR1-hAAT	Human *ApoE/HCR1* and *AAT*	732	−355/+42 hAAT promoter with 326 bp ApoE/HCR1 enhancer	Pompe disease	SPK-3006 (vanglusagene ensiparvovec)	[168,169]
FRE76	Human *ApoE/HCR1* and *AAT*	728	−347/+43 hAAT promoter with 321 bp ApoE/HCR1 enhancer Same as ApoE/HCR1-hAAT	Gaucher disease type 1	FLT201	[170]
LP1	Human *ApoE/HCR1* and *AAT*	448	−212/+43 hAAT promoter with 192 bp ApoE/HCR1 enhancer	Hemophilia B	Hemgenix (AMT-061, CSL222, etranacogene dezaparvovec)	[171,172]
LP1	Human *ApoE/HCR1* and *AAT*	448	−212/+43 hAAT promoter with 192 bp ApoE/HCR1 enhancer	Hemophilia B	scAAV2/8-LP1-hFIXco	[173,174,175,176]
LP1	Human *ApoE/HCR1* and *AAT*	448	−212/+43 hAAT promoter with 192 bp ApoE/HCR1 enhancer	Phenylketonuria (PKU)	HMI-102	[177,178]
LP1	Human *ApoE/HCR1* and *AAT*	448	−212/+43 hAAT promoter with 192 bp ApoE/HCR1 enhancer	Phenylketonuria (PKU)	HMI-103	[160,179]
HLP	Human *ApoE/HCR1* and *AAT*	252	−247/−216 and −143/+43 hAAT promoter with 34 bp ApoE/HCR1	Hemophilia A	AAV-HLP-hFVIII-V3 (GO-8)	[180,181]
HLP	Human *ApoE/HCR1* and *AAT*	252	−247/−216 and −143/+43 hAAT promoter with 34 bp ApoE/HCR1	Hemophilia A	Roctavian (valoctocogene roxaparvovec, BMN 270)	[182,183,184]
FRE1 (HLP2)	Human *ApoE/HCR1* and *AAT*	335	−247/−216 and −143/+43 hAAT promoter with 117 bp ApoE/HCR1 enhancer	Hemophilia B	FLT180a (verbrinacogene setparvovec)	[185,186,187]
FRE1 (HLP2)	Human *ApoE/HCR1* and AAT	335	−247/−216 and −143/+43 hAAT promoter with 117 bp ApoE/HCR1 enhancer	Fabry disease	FLT190	[187,188]
Em-hAATsh	Human *AAT*, synthetic enhancer	139	Shorten −133/+51 hAAT promoter divided on 4 parts with synthetic enhancer composed of hepatocyte TF binding sites	Hemophilia A	ZS802	[189,190]
mTTR mut	Murine *TTR*	223	−138/−135 gact>tgtg mutant mTTR promoter	Hemophilia A	NGGT003	[191]
mTTR mut	Murine *TTR*	223	−138/−135 gact>tgtg mutant mTTR promoter	Hemophilia A	SPK-8011 (dirloctocogene samoparvovec)	[192,193,194]
mTTR enhancer/promoter	Murine *TTR*	330	−204/+5 mTTR promoter with 100 bp mTTR enhancer in antisense orientation	Hemophilia B	AskBio009 (BAX 335)	[195,196]
mTTR enhancer/promoter	Murine *TTR*	330	−204/+5 mTTR promoter with 100 bp mTTR enhancer in antisense orientation	Hemophilia A	TAK-754 (BAX 888)	[197,198,199]
mTTR enhancer/promoter	Murine *TTR*	372	−202/+27 mTTR promoter with modified mTTR enhancer in antisense orientation Shorten ET promoter	Hemophilia B	ANB-002	[200,201]
E03.TTR	Murine and human TTR	296	−189/+1 hTTR promoter with 100 bp mTTR enhancer	Hemophilia A	DTX201 (BAY2599023)	[202,203,204]
E03.TTR	Murine and human *TTR*	290	−189/+1 hTTR promoter with 100 bp mTTR enhancer	Wilson disease	UX701	[205,206]
AlMB2-mTTR482	Murine *TTR*, human *AMBP*	671	−203/+21 mTTR modified promoter with modified 92 bp mTTR enhancer and 2 copies of modified 162 bp hAMBP enhancer	Phenylketonuria (PKU)	SAR444836	[207,208]
CRMSBS2-mTTR	Murine *TTR*, human *AAT*	307	−202/+21 mTTR promoter with modified −122/−51 hAAT in antisense orientation	Hemophilia A	PF-07055480, formerly SB-525 (giroctocogene fitelparvovec)	[209,210,211]
3xCRM8-enTTR-mTTR	Murine *TTR*, human *AAT*	548	−204/+5 mTTR promoter with 100 bp mTTR enhancer and 3 copies of CRM8 (−122/−51 hAAT in antisense orientation)	Hemophilia B	TAK-748 (SHP648)	[196,212]
3xCRM8-enTTR-mTTR	Murine *TTR*, human *AAT*	520	−202/+1 mTTR core promoter with 100 bp mTTR enhancer and 3 copies of CRM8 (−122/−51 hAAT in antisense orientation)	Hemophilia B	VGB-R04	[213]
HCB	*Xenopus laevis Alb*, human *AMBP*	146	−67/−26 xAlb promoter (SynO region) with AbpShort (region of human AMBP shortened to 56 bp), and predicted conservative TSS	Hemophilia A	ASC618	[214,215,216]
GT001 (vector title)	*Xenopus laevis Alb*, canine *AAT*, human *ApoE/HCR1*	266	16 bpHCR1 enhancer with modified canine AAT and −66/+38 Xenopus laevis Alb	Hemophilia A	GS1191-0445	[217,218]
LSP	Human *TBG* and *AMBP*	698	−474/+3 TBG with 2 copies of 101 bp AMBP enhancer	MPS VI (Mucopolysaccharidosis Type VI)	AAV2/8.TBG.hARSB	[219,220]
LSP	Human *TBG* and *AMBP*	747	−475/+4 TBG promoter with 2 copies of modified 98 bp AMBP enhancer with 3 point mutations	Pompe disease	ACTUS-101	[221,222]
LSP	Human *TBG* and *AMBP*	734	−475/+4 TBG promoter with 2 copies of 98 bp AMBP enhancer with 3 point mutations	Hemophilia B	DTX101	[158,223]
LSP *	Human *TBG* and *AMBP*	698	−474/+3 TBG promoter with 2 copies of 101 bp AMBP enhancer	Ornithine Transcarbamylase Deficiency	DTX301	[224,225,226,227]
LXP2.1	Completely synthetic	188	Consists of hepatocyte TF binding sites	Hemophilia A	BBM-H803 (BBM 002)	[228]
LXP2.1	Completely synthetic	188	Consists of hepatocyte TF binding sites	Hemophilia B	BBM-H901	[229]
G6PC1	Human *G6PC1*	2864	−2786/+78 hG6PC1 native promoter	Glycogen storage disease type I (GSDIa)	DTX401	[230,231]
C7	NS	NS	NS	Fabry Disease	AMT-191	[232]

* The DTX301 clinical trial claims to use the TBG promoter despite the presence of two copies of the AMBP enhancer.

## Data Availability

No new data were created or analyzed in this study.

## Supplementary Materials

The following supporting information can be downloaded at: https://www.mdpi.com/article/10.3390/cells15010014/s1, Material S1: List of promoters; Material S2: Supplementary Table S1: Gene therapies with liver-specific promoters [151,152,153,154,155,156,157,158,159,160,161,162,163,164,165,166,167,168,169,170,171,172,173,174,175,176,177,178,179,180,181,182,183,184,185,186,187,188,189,190,191,192,193,194,195,196,197,198,199,200,201,202,203,204,205,206,207,208,209,210,211,212,213,214,215,216,217,218,219,220,221,222,223,224,225,226,227,228,229,230,231,232].
