# Supplementary material for: Engineering Liver-Specific Promoters: A Comprehensive Review of Design, Mechanisms, and Clinical Applications in Gene Therapy
Source: Cells. 2025 Dec 22;15(1):14. doi: 10.3390/cells15010014 (PMC12786076; doi:10.3390/cells15010014)
Supplement: Supplementary file 1 [file cells-15-00014-s001.zip › Supplementary Material S1.pdf]

Supplementary information. List of promoters' sequences used in gene therapy

Legend:

Human AAT promoter

Murine Alb enhancer

Human ApoE/HCR1 enhancer

Synthetic enhancer

Murine TTR promoter

Murine TTR enhancer

Human AMBP enhancer

HNF1 binding site

Xenopus laevis Alb promoter

Conservative TSS

Modified canine AAT promoter

Human TBG promoter

Bold – point mutations

Vertical lines – fragment borders when necessary

VTX801 – hAAT

cgccaccccctccaccttggacacaggacgctgtggtttctgagccaggtacaatgactcctttcggtaagtgcagtggaagctgtacactgcccaggcaaagcgtccgggcagcgtaggcgggcgactcagatcccagccagtggac
ttagcccctgtttgctcctccgataactggggtgaccttggttaatattcaccagcagcctcccccgttgcccctctggatccactgcttaaatacggacgaggacagggccctgtctcctcagcttcaggcaccaccactgacctgggac
agtgaa

rAAV2/5-PBGD – EalbAAT

tcgaggttcctagattacactacacattctgcaagcatagcacagagcaatgttctactttaattactttcattttcttgtatcctcacagcctagaaaataacctgcgttacagcatccactcagtatcccttgagcatgaggtgacacta
cttaacatagggacgagatggtactttgtgtctcctgctctgtcagcagggcacagtacttgctgataccagggaatgtttgttcttaaataccatcattccggacgtgtttgccttggccagttttccatgtacatgcagaaagaagtttg
gactgatcaatacagtcctctgcctttaaagcaataggaaaaggccaacttgtctacgtttagtatgtggctgtagatctgtacccgccacccccctccaccttggacacaggacgctgtggtttctgagccaggtacaatgactcctttcg
gtaagtgcagtggaagctgtacactgcccaggcaaagcgtccgggcagcgtaggcgggcgactcagatcccagccagtggacttagcccctgtttgctcctccgataactggggtgaccttggttaatattcaccagcagcctcccccg
ttgcccctctggatccactgcttaaatacggacgaggacagggccctgtctcctcagcttcaggcacca

SPK-9001 – ApoE/HCR1-hAAT

aggctcagaggcacacaggagtttctgggctcaccctgcccccttccaacccctcagttcccatcctccagcagctgtttgtgtgctgcctctgaagtccacactgaacaaacttcagcctactcatgtccctaaaatgggcaaacattgc
aagcagcaaacagcaaacacacagccctccctgcctgctgaccttggagctggggcagaggtcagagacctctctgggcccatgccacctccaacatccactcgaccccttggaatttcggtggagaggagcagaggttgtcctggc
gtggtttaggtagtgtgagaggggtacccggggatcttgctaccagtggaacagccactaaggattctgcagtgagagcagagggccagctaagtggtactctcccagagactgtctgactcacgccacccccctccaccttggacaca
ggacgctgtggtttctgagccaggtacaatgactcctttcggtaagtgcagtggaagctgtacactgcccaggcaaagcgtccgggcagcgtaggcgggcgactcagatcccagccagtggacttagcccctgtttgctcctccgataa
ctggggtgaccttggttaatattcaccagcagcctcccccgttgcccctctggatccactgcttaaatacggacgaggacagggccctgtctcctcagcttcaggcaccaccactgacctgggacagtgaat

BMN 307 – ApoE/HCR1-hAAT

aggctcagaggcacacaggagtttctgggctcaccctgcccccttccaacccctcagttcccatcctccagcagctgtttgtgtgctgcctctgaagtccacactgaacaaacttcagcctactcatgtccctaaaatgggcaaacattgc
aagcagcaaacagcaaacacacagccctccctgcctgctgaccttggagctggggcagaggtcagagacctctctgggcccatgccacctccaacatccactcgaccccttggaatttcggtggagaggagcagaggttgtcctggc
gtggtttaggtagtgtgagaggggtcgacgatcttgctaccagtggaacagccactaaggattctgcagtgagagcagagggccagctaagtggtactctcccagagactgtctgactcacgccacccccctccaccttggacacagga
cgctgtggtttctgagccaggtacaatgactcctttcggtaagtgcagtggaagctgtacactgcccaggcaaagcgtccgggcagcgtaggcgggcgactcagatcccagccagtggacttagcccctgtttgctcctccgataactg
gggtgaccttggttaatattcaccagcagcctcccccgttgcccctctggatccactgcttaaatacggacgaggacagggccctgtctcctcagcttcaggcaccaccactgacctgggacagtgaatcgtaagta

NGGT002 – ApoE/HCR1-hAAT

aggctcagaggcacacaggagtttctgggctcaccctgcccccttccaacccctcagttcccatcctccagcagctgtttgtgtgctgcctctgaagtccacactgaacaaacttcagcctactcatgtccctaaaatgggcaaacattgc
aagcagcaaacagcaaacacacagccctccctgcctgctgaccttggagctggggcagaggtcagagacctctctgggcccatgccacctccaacatccactcgaccccttggaatttcggtggagaggagcagaggttgtcctggc
gtggtttaggtagtgtgagaggggtcgacgatcttgctaccagtggaacagccactaaggattctgcagtgagagcagagggccagctaagtggtactctcccagagactgtctgactcacgccacccccctccaccttggacacagga

cgctgtggtttctgagccaggtacaatgactcctttcggtaagtgcagtggaagctgtacactgcccaggcaaagcgtccgggcagcgtaggcgggcgactcagatcccagccagtggacttagcccctgtttgctcctccgataactg
gggtgaccttggttaatattcaccagcagcctcccccgttgcccctctggatccactgcttaaatacggacgaggacagggccctgtctcctcagcttcaggcaccaccactgacctgggacagtgaatc

ST-920 – ApoE/HCR1-hAAT

aggctcagaggcacacaggagtttctgggctcaccctgcccccttccaacccctcagttcccatcctccagcagctgtttgtgtgctgcctctgaagtccacactgaacaaacttcagcctactcatgtccctaaaatgggcaaacattgc
aagcagcaaacagcaaacacacagccctccctgcctgctgaccttggagctggggcagaggtcagagacctctctgggcccatgccacctccaacatccactcgaccccttggaatttcggtggagaggagcagaggttgtcctggc
gtggtttaggtagtgtgagaggggtacccggggatcttgctaccagtggaacagccactaaggattctgcagtgagagcagagggccagctaagtggtactctcccagagactgtctgactcacgccacccccctccaccttggacaca
ggacgctgtggtttctgagccaggtacaatgactcctttcggtaagtgcagtggaagctgtacactgcccaggcaaagcgtccgggcagcgtaggcgggcgactcagatcccagccagtggacttagcccctgtttgctcctccgataa
ctggggtgaccttggttaatattcaccagcagcctcccccgttgcccctctggatccactgcttaaatacggacgaggacagggccctgtctcctcagcttcaggcaccaccactgacctgggacagt

GNT0003 – ApoE/HCR1-hAAT

aggctcagaggcacacaggagtttctgggctcaccctgcccccttccaacccctcagttcccatcctccagcagctgtttgtgtgctgcctctgaagtccacactgaacaaacttcagcctactcatgtccctaaaatgggcaaacattgc
aagcagcaaacagcaaacacacagccctccctgcctgctgaccttggagctggggcagaggtcagagacctctctgggcccatgccacctccaacatccactcgaccccttggaatttcggtggagaggagcagaggttgtcctggc
gtggtttaggtagtgtgagaggggtacccggggatcttgctaccagtggaacagccactaaggattctgcagtgagagcagagggccagctaagtggtactctcccagagactgtctgactcacgccacccccctccaccttggacaca
ggacgctgtggtttctgagccaggtacaatgactcctttcggtaagtgcagtggaagctgtacactgcccaggcaaagcgtccgggcagcgtaggcgggcgactcagatcccagccagtggacttagcccctgtttgctcctccgataa
ctggggtgaccttggttaatattcaccagcagcctcccccgttgcccctctggatccactgcttaaatacggacgaggacagggccctgtctcctcagcttcaggcaccaccactgacctgggacagtgaat

SPK-3006 – ApoE/HCR1-hAAT

gccgcaggctcagaggcacacaggagtttctgggctcaccctgcccccttccaacccctcagttcccatcctccagcagctgtttgtgtgctgcctctgaagtccacactgaacaaacttcagcctactcatgtccctaaaatgggcaaa
cattgcaagcagcaaacagcaaacacacagccctccctgcctgctgaccttggagctggggcagaggtcagagacctctctgggcccatgccacctccaacatccactcgaccccttggaatttcggtggagaggagcagaggttgtc
ctggcgtggtttaggtagtgtgagaggggtacccggggatcttgctaccagtggaacagccactaaggattctgcagtgagagcagagggccagctaagtggtactctcccagagactgtctgactcacgccacccccctccaccttgg
acacaggacgctgtggtttctgagccaggtacaatgactcctttcggtaagtgcagtggaagctgtacactgcccaggcaaagcgtccgggcagcgtaggcgggcgactcagatcccagccagtggacttagcccctgtttgctcctcc
gataactggggtgaccttggttaatattcaccagcagcctcccccgttgcccctctggatccactgcttaaatacggacgaggacagggccctgtctcctcagcttcaggcaccaccactgacctgggacagtgaat

FLT201 – FRE76 (ApoE/HCR1-hAAT)

caggctcagaggcacacaggagtttctgggctcaccctgcccccttccaacccctcagttcccatcctccagcagctgtttgtgtgctgcctctgaagtccacactgaacaaacttcagcctactcatgtccctaaaatgggcaaacattg
caagcagcaaacagcaaacacacagccctccctgcctgctgaccttggagctggggcagaggtcagagacctctctgggcccatgccacctccaacatccactcgaccccttggaatttcggtggagaggagcagaggttgtcctgg
cgtggtttaggtagtgtgagaggggtacccggggatcttgctaccagtggaacagccactaaggattctgcagtgagagcagagggccagctaagtggtactctcccagagactgtctgactcacgccacccccctccaccttggacac

aggacgctgtggtttctgagccaggtacaatgactcctttcggtaagtgcagtggaagctgtacactgcccaggcaaagcgtccgggcagcgtaggcgggcgactcagatcccagccagtggacttagcccctgtttgctcctccgata
actggggtgaccttggttaatattcaccagcagcctcccccgttgcccctctggatccactgcttaaatacggacgaggacagggccctgtctcctcagcttcaggcaccaccactgacctggga cagtgaat

AMT-061, scAAV2/8-LP1-hFIXco, HMI-102 and HMI-103 – LP1

ccctaaaatgggcaaacattgcaagcagcaaacagcaaacacacagccctccctgcctgctgaccttggagctggggcagaggtcagagacctctctgggcccatgccacctccaacatccactcgaccccttggaatttcggtgga
gaggagcagaggttgtcctggcgtggtttaggtagtgtgagaggggaatgactcctttcggtaagtgcagtggaagctgtacactgcccaggcaaagcgtccgggcagcgtaggcgggcgactcagatcccagccagtggacttagc
ccctgtttgctcctccgataactggggtgaccttggttaatattcaccagcagcctcccccgttgcccctctggatccactgcttaaatacggacgaggacagggccctgtctcctcagcttcaggcaccaccactgacctgggacagtga
atc

BMN 270 and AAV-HLP-hFVIII-V3 (GO-8) – HLP

tgtttgctgcttgcaatgtttgcccattttagggtggacacaggacgctgtggtttctgagccaggggcgactcagatcccagccagtggacttagcccctgtttgctcctccgataactggggtgaccttggttaatattcaccagcagc
ctcccccgttgcccctctggatccactgcttaaatacggacgaggacagggccctgtctcctcagcttcaggcaccaccactgacctgggacagtgaatc

FLT180a and FLT190 – HLP2 (FRE1)

ccctaaaatgggcaaacattgcaagcagcaaacagcaaacacacagccctccctgcctgctgaccttggagctggggcagaggtcagacacctctctgggcccatgccacctccaactggacacaggacgctgtggtttctgagcca
ggggggcgactcagatcccagccagtggacttagcccctgtttgctcctccgataactggggtgaccttggttaatattcaccagcagcctcccccgttgcccctctggatccactgcttaaatacgga cgaggacagggccctgtctcctc
agcttcaggcaccaccactgacctgggacagtgaatc

ZS802 – Em-hAATsh

tggactttgcactattgcacaattgtaaacagttaatcattaacattacgtaacttaaggcagcgtaggcgggagtggacttagcccctgtggttaatattcaccagcagcctcccccgttgcccctcactgacctggg

SPK-8011 and NGGT003 – mTTR mut

gtctgtctgcacatttcgtagagcgagtgttccgatactctaatctccctaggcaaggttcatatt**gact**taggttacttattctccttttgttgactaagtcaataatcagaatcagcaggtttggagtcagcttggcagggatcagcagcc
tgggttggaaggagggggtataaaagccccttcaccaggagaagccgtcacacagatccacaagctcctg

BAX 335 and BAX 888 (TAK-754) – mTTR enhancer/promoter (enTTR-mTTR)

cgagggcactgggaggatgttgagtaagatggaaaactactgatgacccttgcagagacagagtattaggacatgtttgaacaggggccgggcgatcagcaggtagctctagaggatccccgtctgtctgcacatttcgtagagcgagtgttccgatactctaatctccctaggcaaggttcatatttgtgtaggttacttattctccttttgttgactaagtcaataatcagaatcagcaggtttggagtcagcttggcagggatcagcagcctgggttggaaggagggggtataaaagccccttcaccaggagaagccgtcacacaga

ANB-002 – modified mTTR enhancer/promoter

tcgagcttgggctgcaggtcgagggcactgggaggatgttgagtaagatggaaaactactgatgacccttgcagagacagagtattaggacatgtttgaacaggggccggcgatcagcaggtagctctagaggatccccgtctgtctgcacatttcgtagagcgagtgttccgatactctaatctccctaggcaaggttcatatttgtgtaggttacttattctccttttgttgactaagtcaataatcagaatcagcaggtttggagtcagcttggcagggatcagcagcctgggttggaaggagggggtataaaagccccttcaccaggagaagccgtcacacagatccacaagctcctgacaggaagct

DTX201 (BAY2599023) – E03.TTR

ctacctcgtgatcgcccggcccctgttcaaacatgtcctaatactctgtctctgcaagggtcatcagtagttttccatcttactcaacatcctcccagtggaattcatttcatagaacgaatgttccgatgctctaatctctctagacaaggttcatatttgtatgggttacttattctctctttgttgactaagtcaataatcagaatcagcaggtttgcagtcagattggcagggataagcagcctagctcaggagaagtgagtataaaagccccaggctgggagcagccatca

UX701 – E03.TTR

ctacctcgtgatcgcccggcccctgttcaaacatgtcctaatactctgtctctgcaagggtcatcagtagttttccatcttactcaacatcctcccagtgatttcatagaacgaatgttccgatgctctaatctctctagacaaggttcatatttgtatgggttacttattctctctttgttgactaagtcaataatcagaatcagcaggtttgcagtcagattggcagggataagcagcctagctcaggagaagtgagtataaaagccccaggctgggagcagccatca

SAR444836 – AlMB2-mTTR482

ggccccaggttaatttttaaaaagcagtcaaa**gg**tc**a**aagtggcccttggcagcatttactctctct**a**tt**ga**ct**t**tggttaataatctcaggagcacaaacattcctggaggcaggagaagaaatcaacatcctggacttatcctctgggcctctccccaccttcgatggccccaggttaatttttaaaaagcagtcaaa**gg**tc**a**aagtggcccttggcagcatttactctctct**a**tt**g**act**t**tggttaataatctcaggagcacaaacattcctggaggcaggagaagaaatcaacatcctggacttatcctctgggcctctccccaccgatatctacctgctgatcgcccggcccctgttcaaacatgtcctaatactctgtc**gggg**caa**a**ggtc**gg**cagtagttttccatcttactcaacatcctcccagtgtacgtaggatcctgtctgtctgcacatttcgtagagcgagtgttccgatactctaatctccc**gg**ggcaa**a**gg**t**c**g**tatt**gact**taggttacttattctccttttgttgactaagtcaataatcagaatcagcaggtttggagtcagcttggcagggatcagcagcctgggttggaaggagggggtataaaagccccttcaccaggagaagccgtcacacagatccacaagctcctg

PF-07055480 (SB-525) – CRMSBS2-mTTR

gggggaggctgctggtgaatattaaccaag**a**tcaccccagtta**c**cggaggagcaaacaggg**a**ctaagtt**t**cacacgcgtggtaccgtctgtctgcacatttcgtagagcgagtgttccgatactctaatctccctaggcaaggttcatat
ttgtgtaggttacttattctccttttgttgactaagtcaataatcagaatcagcaggtttggagtcagcttggcagggatcagcagcctgggttggaaggagggggtataaaagccccttcaccaggagaagccgtcacacagatccac
aagctcctg

TAK-748 – 3xCRM8-enTTR-mTTR

gggggaggctgctggtgaatattaaccaaggtcaccccagttatcggaggagcaaacaggggctaagtccaccgggggaggctgctggtgaatattaaccaaggtcaccccagttatcggaggagcaaacaggggctaagtccac
cgggggaggctgctggtgaatattaaccaaggtcaccccagttatcggaggagcaaacaggggctaagtccaccgagggcactgggaggatgttgagtaagatggaaaactactgatgacccttgcagagacagagtattaggac
atgtttgaacaggggccgggcgatcagcaggtagctctagaggatccccgtctgtctgcacatttcgtagagcgagtgttccgatactctaatctccctaggcaaggttcatatttgtgtaggttacttattctccttttgttgactaagtca
ataatcagaatcagcaggtttggagtcagcttggcagggatcagcagcctgggttggaaggagggggtataaaagccccttcaccaggagaagccgtcacacaga

VGB-R04 – 3xCRM8-enTTR-mTTR

gggggaggctgctggtgaatattaaccaaggtcaccccagttatcggaggagcaaacaggggctaagtccaccgggggaggctgctggtgaatattaaccaaggtcaccccagttatcggaggagcaaacaggggctaagtccac
cgggggaggctgctggtgaatattaaccaaggtcaccccagttatcggaggagcaaacaggggctaagtccaccactgggaggatgttgagtaagatggaaaactactgatgacccttgcagagacagagtattaggacatgtttg
aacaggggccgggcgatcagcaggtaggtctgtctgcacatttcgtagagcgagtgttccgatactctaatctccctaggcaaggttcatatttgtgtaggttacttattctccttttgttgactaagtcaataatcagaatcagcaggttt
ggagtcagcttggcagggatcagcagcctgggttggaaggagggggtataaaagccccttcaccaggagaagccgtc

ASC618 – HCB

gttaatcattaagtcgttaatttttgtggcccttgcgatgtttgctctggttaataatctcaggacaaacagaggttaataattttccagatctctctgagcaatagtataaaaggccagcagcagcctgaccacatctcatcctc

GS1191-0445 – GT001 (vector title)

gctaaaatgggcaaacatgctgtttactgagctgggcacaatgacctttggcgagctggacagagggccgggtgctgacgtgcaggcgggtgggctgacctttaaacttgtttgctcctccgataaccggggtgaccttggttaatcatt
aaccagcaatgggggttaataattttccagatctctctgagcaatagtataaaacaagaggtatcactcatttcagatcaggcttctcagaggtccccacccaatacatctccagtc

AAV2/8.TBG.hARSB and DTX301 – LSP

caggttaatttttaaaaagcagtcaaaagtccaagtggcccttggcagcatttactctctctgtttgctctggttaataatctcaggagcacaaacattccagatccaggttaatttttaaaaagcagtcaaaagtccaagtggcccttgg
cagcatttactctctctgtttgctctggttaataatctcaggagcacaaacattccagatccggcgcgccagggctggaagctacctttgacatcatttcctctgcgaatgcatgtataatttctacagaacctattagaaaggatcaccca
gcctctgcttttgtacaactttcccttaaaaaactgccaattccactgctgtttggcccaatagtgagaactttttcctgctgcctcttggtgcttttgcctatggcccctattctgcctgctga agacactcttgccagcatggacttaaaccc
ctccagctctgacaatcctctttctcttttgttttacatgaagggtctggcagccaaagcaatcactcaaagttcaaaccttatcatttttgctttgttcctcttggccttggttttgtacatcagctttgaaaataccatcc cagggttaatgc
tggggttaatttataactaagagtgctctagttttgcaatacaggacatgctataaaaatggaaagatgttgctttctgagaga

ACTUS-101 – LSP

gttaatttttaaaaagcagtcaaaagtccaagtgcccttg**cg**agcatttactctctctgtttgctctggttaataatctcaggagcacaaacattccttactagttctaggagttaatttttaaaaagcagtcaaaagtccaagtgcccttg
cgagcatttactctctctgtttgctctggttaataatctcaggagcacaaacattcctactagttctagagcggccgccagtgtgctggaattcggctttttagggctggaagctacctttgacatcatttcctctgcgaatgcatgtataa
tttctacagaacctattagaaaggatcacccagcctctgcttttgtacaactttcccttaaaaaactgccaatcccactgctgtttggcccaatagtgagaactttttcctgctgcctcttggtg cttttgcctatggcccctattctgcctgct
gaagacactcttgccagcatggacttaaacccctccagctctgacaatcctctttctcttttgttttacatgaagggtctggcagccaaagcaatcactcaaagttcaaaccttatcatttttgctttgttcctcttggccttggttttgtaca
tcagctttgaaaataccatcccagggttaatgctggggttaatttataactgagagtgctctagttctgcaatacaggacatgctataaaaatggaaagatgttgctttctgagagatcagctta catgtg

DTX101 – LSP

gttaatttttaaaaagcagtcaaaagtccaagtgcccttgcgagcatttactctctctgtttgctctggttaataatctcaggagcacaaacattccttactagttctaggagttaatttttaaaaagcagtcaaaagtccaagtgcccttg
cgagcatttactctctctgtttgctctggttaataatctcaggagcacaaacattcctactagttctagagcggccgccagtgtgctggaattcggctttttagggctggaagctacctttgacatcatttcctctgcgaatgcatgtataa
tttctacagaacctattagaaaggatcacccagcctctgcttttgtacaactttcccttaaaaaactgccaatcccactgctgtttggcccaatagtgagaactttttcctgctgcctcttggtg cttttgcctatggcccctattctgcctgct
gaagacactcttgccagcatggacttaaacccctccagctctgacaatcctctttctcttttgttttacatgaagggtctggcagccaaagcaatcactcaaagttcaaaccttatcatttttgctttgttcctcttggccttggttttgtaca
tcagctttgaaaataccatcccagggttaatgctggggttaatttataactgagagtgctctagttctgcaatacaggacatgctataaaaatggaaagatgttgctttctgagagat

BBM-H901 and BBM-H803 – LXP2.1

aggttaatttttaaaaagcagtcaaaagtccaagtggcccttggcagcatttactctctctgtttactctggttaataatctcaggagtacaaacattccagatccaggttaatttttaaaaaaag agtgctctagttttgcaatacaggac
atgctataaaaagcgaagcgcgcggtgggcggggtt

DTX401 – native human G6PC1 promoter

cctttgagaatccacggtgtctcgatgcagtcagctttctaacaagctggggcctcacctgttttcccacggataaaaacgtgctggaggaagcagaaaggggctggcaggtggaaagatgaggaccagctcatcgtctcatgactat
gaggttgctctgatccagagggtcccccctgcctggtggcccaccgccaggaagactcccactgtccctggatgcccagagtgggatgtcaactccatcacttatcaactccttatccataggggtattcttcctgaggcgtctcagaaaa
cagggccctccccatatgctgaccacataatagaacccctcccaactcagagaccctggctgctagctgccctggcatgacccagacagtggcctttgtatatgtttttagactcaccttgactcacctctgaccatagaaactctcatcc
cagaggtcactgcaatagttactccacaacagaggcttatctgggtagagggaggctccctacctatggcccagcagccctgacagtgcagatcacatataccccacgccccagcactgcctgccacgcatgggcttactttacaccc
acccacagtcaccaacacattacctgctctccaaggttaggcgtggcaggagaagtttgcttggaccagcagaaaccatgcagtcaaggacaactggagtcagcatgggctgggtgcgagcccttggtggggtggggaggagactc
caggtcatacctcctggaggatgttttaatcatttccagcatggaatgctgtcaacttttgccacagattcattagctctgagtttctttttctgtccccagctaccccttacatgtcaatatggacttaatgatgggaaattcaggcaagtt
tttaaacattttattccccctggctcttatcctcaaaaaatgcatgaatttggaggcagtggctcatgcctgtaatcccaatgctttgctaggttgaggcgggaggatcacttgaagccaggaatttgagaccagcctgggccgcatagtg
agaccccgtttctacaaaaataaataaataaataataaataatagtgatatgaagcatgattaaatagccctatttttttaaaatgcatgagttcgttacctgattcattccctggttcctttcacagtcctccgtgacccaagtgttagggt
tttggtctctctactatttgtaggctgatatatagtatacacacacacacacacacatatacacacacacagtgtatcttgagctttcttttgtatatctacacacatatgtataagaaagctcaagatatagaagcccttttttcaaaaa
taactgaaagtttcaaactctttaagtctccagttaccattttgctggtattcttatttggaaccatacattcatcatattgttgcacagtaagactatacattcattattttgcttaaacgtatgagttaaaacacttggccaggcatggtgg
ttcacacctgtaatcccagagctttgggaagccaagactggcagatctcttgagctcaggaattcaagaccagcctgggcaacatggaaaaaccccatctctacaaaagatagaaaaattagccaggcatggtggcgtgtgcctgtg
gtcccagctactcaggaggctgaggtgggaggatcacattagcccaggaggttgaggctgcagtgagccgtgattatgccactgcactccagcctgggagacagagtgagaccctgtttcaaaaaaaagagagagaaaatttaaaa
aagaaaacaacaccaagggctgtaactttaaggtcattaaatgaattaatcactgcattcaaaaacgattactttctggccctaagagacatgaggccaataccaggaagggggttgatctcccaaaccagaggcagaccctagact
ctaatacagttaaggaaagaccagcaagatgatagtccccaatacaatagaagttactatattttatttgttgtttttcttttgttttgttttgttttgttttgttttgttttagagactggggtcttgctcgattgcccaggctgtagtgcagcg
gtgggacaatagctcactgcagactccaactcctgggctcaagcaatcctcctgcctcagcctcctgaatagctgggactacaagggtacaccatcacacacaccaaaacaatttttttaaatttttgtgtagaaacgagggtcttgcttt
gttgcccaggctggtctccaactcctggcttcaagggatcctcccacctcagcctcccaaattgctgggattacaggtgtgagccaccacaaccagccagaactttactaattttaaaattaagaacttaaaacttgaatagctagagca
ccaagatttttctttgtccccaaataagtgcagttgcaggcatagaaaatctgacatctttgcaagaatcatcgtggatgtagactctgtcctgtgtctctggcctggtttcggggaccaggagggcagacccttgcactgccaagaagc
atgccaaagttaatcattggccctgctgagtacatggccgatcaggctgtttttgtgtgcctgtttttctattttacgtaaatcaccctgaacatgtttgcatcaacctactggtgatgcacctttgatcaatacattttagacaaacgtggtt
tttgagtccaaagatcagggctgggttgacctgaatactggatacagggcatataaaacaggggcaaggcacagactcatagcagagcaatcaccaccaagcctggaataactgcaagggctctgctgacatcttcctgaggtgcca
aggaaatgagg
