# Supplementary material for: Engineering Liver-Specific Promoters: A Comprehensive Review of Design, Mechanisms, and Clinical Applications in Gene Therapy
Source: Cells. 2025 Dec 22;15(1):14. doi: 10.3390/cells15010014 (PMC12786076; doi:10.3390/cells15010014)
Supplement: Supplementary file 1 [file cells-15-00014-s001.zip › Supplementary Material S2.pdf]

Supplementary Table S1. Gene therapies with liver-specific promoters

Promoter	Gene of origin	Length, bp	Features	Disease	Gene therapy	Developer	Vector	Clinical trial	Stage	Ref.
hAAT	Human *AAT*	305	−264/+41 hAAT promoter	Wilson disease	VTX801	Vivet Therapeutics	AAV3B	NCT04537377	Phase 1-2 active, not recruiting	[151–153]
EalbAAT	Human *AAT*, murine *Alb*	673	−264/+20 hAAT promoter with 376 bp mAlb enhancer	AIP (acute intermittent porphyria)	rAAV2/5-PBGD	Digna Biotech S.L.	AAV5	NCT02082860	Phase 1 completed	[154–156]
ApoE/HCR 1-hAAT	Human *ApoE/HCR 1* and *AAT*	727	−355/+42 hAAT promoter with 321 bp ApoE/HCR1 enhancer	Hemophilia B	Beqvez (fidanacogene elaparvovec, SPK-9001)	Pfizer	AAVrh74var (AAV-Spark100)	NCT05568719	Phase 3 (FDA approved)	[157,158]
ApoE/HCR 1-hAAT	Human *ApoE/HCR 1* and *AAT*	732	−353/+50 hAAT promoter with 321 bp ApoE/HCR1 enhancer	Phenylketonuria (PKU)	BMN 307	BioMarin Pharmaceutical	AAV5	NCT04480567	Phase 1/2 active, not recruiting	[159,160]
ApoE/HCR 1-hAAT	Human *ApoE/HCR 1* and *AAT*	725	−355/+43 hAAT promoter with 321 bp ApoE/HCR1 enhancer	Phenylketonuria (PKU)	NGGT002	NGGT INC.	AAV8	NCT06687733	Phase 1/2 recruiting	[161]
ApoE/HCR 1-hAAT	Human *ApoE/HCR 1* and *AAT*	723	−355/+38 hAAT promoter with 321 bp ApoE/HCR1 enhancer	Fabry Disease	ST-920	Sangamo Therapeutics	AAV6	NCT04046224	Phase 1/2 completed	[162–164]
ApoE/HCR 1-hAAT	Human *ApoE/HCR 1* and *AAT*	727	−355/+42 hAAT promoter with 321 bp ApoE/HCR1 enhancer	Crigler-Najjar syndrome	GNT0003	Genethon	AAV8	NCT03466463	Recruiting	[165–167]
ApoE/HCR 1-hAAT	Human *ApoE/HCR 1* and *AAT*	732	−355/+42 hAAT promoter with 326 bp ApoE/HCR1 enhancer	Pompe disease	SPK-3006 (vanglusagene ensiparvovec)	Spark Therapeutics, Inc.	AAVrh74var (AAV-Spark100)	NCT04093349	Phase 1/2 active, not recruiting	[168,169]
FRE76	Human *ApoE/HCR 1* and *AAT*	728	−347/+43 hAAT promoter with 321 bp ApoE/HCR1 enhancer Same as ApoE/HCR1-hAAT	Gaucher disease type 1	FLT201	Freeline Therapeutics, Ltd/Spur Therapeutics	AAVS3 (AAV8 and AAV3B chimera)	NCT07223944	Phase 3	[170]
LP1	Human *ApoE/HCR 1* and *AAT*	448	−212/+43 hAAT promoter with 192 bp ApoE/HCR1 enhancer	Hemophilia B	Hemgenix (AMT-061, CSL222, etranacogene dezaparvovec)	UniQure/CSL Behring	AAV5	NCT07080905	Phase 3 (FDA approved)	[171,172]

LP1	Human *ApoE/HCR 1* and *AAT*	448	−212/+43 hAAT promoter with 192 bp ApoE/HCR1 enhancer	Hemophilia B	scAAV2/8-LP1-hFIXco	St. Jude Children's Research Hospital	scAAV8	NCT00979238	Phase 1 active, not recruiting	[173–176]
LP1	Human *ApoE/HCR 1* and *AAT*	448	−212/+43 hAAT promoter with 192 bp ApoE/HCR1 enhancer	Phenylketonuria (PKU)	HMI-102	Homology Medicines, Inc	AAVHSC15	NCT03952156	Phase 1/2 terminated	[177,178]
LP1	Human *ApoE/HCR 1* and *AAT*	448	−212/+43 hAAT promoter with 192 bp ApoE/HCR1 enhancer	Phenylketonuria (PKU)	HMI-103	Homology Medicines, Inc	AAVHSC15	NCT05222178	Phase 1 terminated	[179,180]
HLP	Human *ApoE/HCR 1* and *AAT*	252	−247/−216 and −143/+43 hAAT promoter with 34 bp ApoE/HCR1	Hemophilia A	AAV-HLP-hFVIII-V3 (GO-8)	University College, London	AAV8	NCT03001830	Phase 1/2 active, not recruiting	[181,182]
HLP	Human *ApoE/HCR 1* and *AAT*	252	−247/−216 and −143/+43 hAAT promoter with 34 bp ApoE/HCR1	Hemophilia A	Roctavian (valoctocogene roxaparvovec, BMN 270)	BioMarin Pharmaceutical	AAV5	NCT03370913	Phase 3 completed (FDA approved)	[183–185]
FRE1 (HLP2)	Human *ApoE/HCR 1* and *AAT*	335	−247/−216 and −143/+43 hAAT promoter with 117 bp ApoE/HCR1 enhancer	Hemophilia B	FLT180a (verbrinacogene setparvovec)	Freeline Therapeutics, Ltd/Spur Therapeutics	AAVS3 (AAV8 and AAV3B chimera)	NCT05164471	Phase 1/2 terminated	[186–188]
FRE1 (HLP2)	Human *ApoE/HCR 1* and *AAT*	335	−247/−216 and −143/+43 hAAT promoter with 117 bp ApoE/HCR1 enhancer	Fabry disease	FLT190	Freeline Therapeutics, Ltd/Spur Therapeutics	AAVS3 (AAV8 and AAV3B chimera)	NCT04040049	Phase 1/2 terminated	[188,189]
Em-hAATsh	Human *AAT*, synthetic enhancer	139	Shorten −133/+51 hAAT promoter divided on 4 parts with synthetic enhancer composed of hepatocyte TF binding sites	Hemophilia A	ZS802	Sichuan Real & Best Biotech Co., Ltd./Institute of Hematology & Blood Diseases Hospital	AAV5	NCT05523128, CTR20232175	Recruiting	[190,191]
mTTR mut	Murine *TTR*	223	−138/−135 gact>tgtg mutant mTTR promoter	Hemophilia A	NGGT003	Suzhou Nuojiebei Biotechnology Co./Institute of Hematology & Blood Diseases Hospital	AAV8	NCT06238908	Recruiting	[192]
mTTR mut	Murine *TTR*	223	−138/−135 gact>tgtg mutant mTTR promoter	Hemophilia A	SPK-8011 (dirloctocogene samoparvovec)	Spark Therapeutics, Inc.	SPK200 (derived from LK03)	NCT06297486	Phase 3 withdrawn	[193–195]

mTTR enhancer/promoter	Murine *TTR*	330	−204/+5 mTTR promoter with 100 bp mTTR enhancer in antisense orientation	Hemophilia B	AskBio009 (BAX 335)	Baxalta/Takeda	scAAV8	NCT01687608	Phase 1/2 active, not recruiting	[196,197]
mTTR enhancer/promoter	Murine *TTR*	330	−204/+5 mTTR promoter with 100 bp mTTR enhancer in antisense orientation	Hemophilia A	TAK-754 (BAX 888)	Baxalta/Takeda	AAV8	NCT03370172	Phase 1/2 completed	[198–200]
mTTR enhancer/promoter	Murine *TTR*	372	−202/+27 mTTR promoter with modified mTTR enhancer in antisense orientation	Hemophilia B	ANB-002	BIOCAD	AAV5	NCT06700096	Phase 3	[201,202]
E03.TTR	Murine and human TTR	296	Shorten ET promoter −189/+1 hTTR promoter with 100 bp mTTR enhancer	Hemophilia A	DTX201 (BAY2599023)	Ultragenyx Pharmaceutical Inc/Bayer	AAVhu37	NCT03588299	Phase 1/2 active, not recruiting	[203–205]
E03.TTR	Murine and human *TTR*	290	−189/+1 hTTR promoter with 100 bp mTTR enhancer	Wilson disease	UX701	Ultragenyx Pharmaceutical Inc	AAV9	NCT04884815	Phase 1/2 active, not recruiting	[206,207]
AIMB2-mTTR482	Murine *TTR*, human *AMBP*	671	−203/+21 mTTR modified promoter with modified 92 bp mTTR enhancer and 2 copies of modified 162 bp hAMBP enhancer	Phenylketonuria (PKU)	SAR444836	Sanofi	AAVSNY001	NCT05972629	Phase 1/2 active, not recruiting	[208,209]
CRMSBS2-mTTR	Murine *TTR*, human *AAT*	307	−202/+21 mTTR promoter with modified −122/−51 hAAT in antisense orientation	Hemophilia A	PF-07055480, formerly SB-525 (giroctocogene fitelparvovec)	Sangamo Therapeutics/Pfizer	AAV6	NCT04370054	Phase 3 active, not recruiting	[210–212]
3xCRM8-enTTR-mTTR	Murine *TTR*, human *AAT*	548	−204/+5 mTTR promoter with 100 bp mTTR enhancer and 3 copies of CRM8 (−122/−51 hAAT in antisense orientation)	Hemophilia B	TAK-748 (SHP648)	Baxalta/Takeda	AAV8	NCT04394286	Phase 1/2 terminated	[213,214]
3xCRM8-enTTR-mTTR	Murine *TTR*,	520	−202/+1 mTTR core promoter with 100 bp mTTR enhancer and 3	Hemophilia B	VGB-R04	Shanghai Vitalgen BioPharma	AAV8	NCT05441553	Early phase 1	[215]

	human *AAT*		copies of CRM8 (−122/−51 hAAT in antisense orientation)							
HCB	*Xenopus laevis Alb*, human *AMBP*	146	−67/−26 xAlb promoter (SynO region) with AbpShort (region of human AMBP shortened to 56 bp), and predicted conservative TSS	Hemophilia A	ASC618	ASC Therapeutics	AAV8	NCT04676048	Phase 1/2 recruiting	[216–218]
GT001 (vector title)	*Xenopus laevis Alb*, canine *AAT*, human *ApoE*/*HCR 1*	266	16 bp HCR1 enhancer with modified canine AAT and −66/+38 Xenopus laevis Alb	Hemophilia A	GS1191-0445	Gritgen Therapeutics Co., Ltd.	AAV8	NCT06833983	Phase 3 recruiting	[219,220]
LSP	Human *TBG* and *AMBP*	698	−474/+3 TBG with 2 copies of 101 bp AMBP enhancer	MPS VI (Mucopolysaccharidosis Type VI)	AAV2/8.TBG.hARSB	Fondazione Telethon	AAV8	NCT03173521	Phase 1/2 completed	[221,222]
LSP	Human *TBG* and *AMBP*	747	−475/+4 TBG promoter with 2 copies of modified 98 bp AMBP enhancer with 3 point mutations	Pompe disease	ACTUS-101	AskBio Inc	AAV8	NCT03533673	Phase 1-2 active, not recruiting	[223,224]
LSP	Human *TBG* and *AMBP*	734	−475/+4 TBG promoter with 2 copies of 98 bp AMBP enhancer with 3 point mutations	Hemophilia B	DTX101	Ultragenyx Pharmaceutical Inc	AAVrh10	NCT02618915	Phase 1/2 terminated	[225,226]
LSP*	Human *TBG* and *AMBP*	698	−474/+3 TBG promoter with 2 copies of 101 bp AMBP enhancer	Ornithine Transcarbamylase Deficiency	DTX301	Ultragenyx Pharmaceutical Inc	scAAV8	NCT05345171	Phase 3 active, not recruiting	[227–230]
LXP2.1	Completely synthetic	188	Consists of hepatocyte TF binding sites	Hemophilia A	BBM-H803 (BBM 002)	Shanghai Xinzhi BioMed Co., Ltd.	AAV843 (AAVXL32.1)	NCT06111638	Phase 1/2 recruiting	[231]
LXP2.1	Completely synthetic	188	Consists of hepatocyte TF binding sites	Hemophilia B	BBM-H901	Shanghai Xinzhi BioMed Co., Ltd.	scAAV843 (XL32.1)	NCT05203679	Phase 2/3 (Approved in China)	[232]

G6PC1	Human *G6PC1*	2864	−2786/+78 hG6PC1 native promoter	Glycogen storage disease type I (GSDIa)	DTX401	Ultragenyx Pharmaceutical Inc	AAV8	NCT03517085	Phase 3 active, not recruiting	[233,234]
C7	NS	NS	NS	Fabry Disease	AMT-191	UniQure Biopharma B.V.	AAV5	NCT06270316	Phase 1/2 recruiting	[235]

*The DTX301 clinical trial claims to use the TBG promoter despite the presence of two copies of the AMBP enhancer.
